# Phenotypic variation and quantitative trait loci for resistance to southern anthracnose and clover rot in red clover

**DOI:** 10.1007/s00122-022-04223-8

**Published:** 2022-09-25

**Authors:** Lea A. Frey, Tim Vleugels, Tom Ruttink, Franz X. Schubiger, Marie Pégard, Leif Skøt, Christoph Grieder, Bruno Studer, Isabel Roldán-Ruiz, Roland Kölliker

**Affiliations:** 1 Molecular Plant Breeding, Institute of Agricultural Sciences, ETH Zurich, 8092 Zurich, Switzerland; 2 Flanders Research Institute for Agriculture, Fisheries and Food (ILVO), Plant Sciences Unit, Caritasstraat 39, 9090 Melle, Belgium; 3 Agroscope, Plant Breeding, Reckenholzstrasse 191, 8046 Zurich, Switzerland; 4 INRAE, Centre Nouvelle-Aquitaine-Poitiers, UR4 (UR P3F), 86600 Lusignan, France; 5 Institute of Biological, Environmental & Rural Sciences, Aberystwyth University, Gogerddan, Aberystwyth, Ceredigion SY23 3EE UK; 6 Department of Plant Biotechnology and Bioinformatics, Ghent University, Ghent, Belgium

## Abstract

**Key message:**

High variability for and candidate loci associated with resistance to southern anthracnose and clover rot in a worldwide collection of red clover provide a first basis for genomics-assisted breeding.

**Abstract:**

Red clover (*Trifolium pratense* L.) is an important forage legume of temperate regions, particularly valued for its high yield potential and its high forage quality. Despite substantial breeding progress during the last decades, continuous improvement of cultivars is crucial to ensure yield stability in view of newly emerging diseases or changing climatic conditions. The high amount of genetic diversity present in red clover ecotypes, landraces, and cultivars provides an invaluable, but often unexploited resource for the improvement of key traits such as yield, quality, and resistance to biotic and abiotic stresses. A collection of 397 red clover accessions was genotyped using a pooled genotyping-by-sequencing approach with 200 plants per accession. Resistance to the two most pertinent diseases in red clover production, southern anthracnose caused by *Colletotrichum trifolii*, and clover rot caused by *Sclerotinia trifoliorum,* was assessed using spray inoculation. The mean survival rate for southern anthracnose was 22.9% and the mean resistance index for clover rot was 34.0%. Genome-wide association analysis revealed several loci significantly associated with resistance to southern anthracnose and clover rot. Most of these loci are in coding regions. One quantitative trait locus (QTL) on chromosome 1 explained 16.8% of the variation in resistance to southern anthracnose. For clover rot resistance we found eight QTL, explaining together 80.2% of the total phenotypic variation. The SNPs associated with these QTL provide a promising resource for marker-assisted selection in existing breeding programs, facilitating the development of novel cultivars with increased resistance against two devastating fungal diseases of red clover.

**Supplementary Information:**

The online version contains supplementary material available at 10.1007/s00122-022-04223-8.

## Introduction

Red clover (*Trifolium pratense* L.), one of the most important forage legumes in temperate climates, is grown in mixture with forage species or as a pure stand (Taylor 2008). Red clover is appreciated for its high forage yield (up to 14 tons dry matter/ha/year), its high protein content and digestibility, its ability to fix atmospheric nitrogen, and its beneficial effects on soil structure (Broderick 1995; Taylor and Quesenberry 1996; Halling et al. 2004; Nyfeler et al. 2011). Red clover is an outcrossing species with a high degree of self-incompatibility and a genome size of approximately 420 Mb (2*n* = 2*x* = 14; De Vega et al. 2015). Main breeding objectives are a high and stable forage yield, persistence, and good forage quality. Disease and insect resistance are important aspects of red clover breeding programs and necessary to meet the requirements of a successful cultivar (Taylor 2008; Boller et al. 2010).

Key fungal pathogens threatening European red clover production and leading to severe yield losses are *Colletotrichum trifolii* Bain & Essary, causing southern anthracnose, and *Sclerotinia trifoliorum* Erikks, causing clover rot. *C. trifolii* was first described in 1906 and has since been reported on a regular basis in most European countries (Bain and Essary 1906; Schubiger et al. 2004; Jacob et al. 2015). In the southern parts of the USA, where the disease has long been a major problem, intensive breeding efforts led to largely resistant cultivars (Taylor 2008). *C. trifolii* has benefitted from warmer summer temperatures in Central Europe, and southern anthracnose has become a limiting factor for red clover production, increasing the demand for resistant cultivars (Boller et al. 2010). *C. trifolii* is a hemibiotrophic fungus that mainly spreads by rain and wind and causes brown coloration on petioles and stems of red clover. Once the xylem is infected the plant begins to shrivel, stem lesions occur, and the plant eventually dies off (De Silva et al. 2017). While the genetics of southern anthracnose resistance in red clover remains largely unknown, resistance to *Colletotrichum* spp. has been extensively studied in other plant species, including soybean (*Glycine max* L.), common bean (*Phaseolus vulgaris* L.), and alfalfa (*Medicago sativa* L.), as reviewed in Dean et al. (2012).

Clover rot, also known as *Sclerotinia* crown, stem rot, or clover cancer is caused by the necrotrophic fungus *S. trifoliorum*, which can survive up to 7 years as soil-borne resting bodies (sclerotia). In autumn, sclerotia develop apothecia, which release airborne ascospores that infect red clover leaves and slowly colonize the whole plant during winter (Taylor and Quesenberry 1996; Öhberg 2008). Prolonged conditions of high humidity such as temperate, damp weather, or long periods of snow cover favor clover rot development (Saharan and Mehta 2010). Although little is known on its genetics, resistance to clover rot in red clover is assumed to be a quantitative trait (Poland et al. 2009; Klimenko et al. 2010; Vleugels and Van Bockstaele 2013).

As southern anthracnose and clover rot can cause substantial losses in European red clover production, resistance breeding is of prime importance. Different aspects need to be considered in developing resistant cultivars. First, as for most diseases, natural infection typically varies between years and between locations. Therefore, infection in breeding trials is rarely homogeneous, and disease development strongly depends on weather conditions. Second, most red clover cultivars are bred as synthetic, population-based varieties, complicating the fixation of resistance alleles. Third, little is known on the genetic basis of resistance against southern anthracnose or clover rot, precluding the use of molecular markers in resistance breeding. Resistance breeding for southern anthracnose, and for clover rot to a lesser extent, has been relatively successful when using artificial inoculations or bio-tests in controlled environments (Marum et al. 1994; Delclos and Duc 1996; Schubiger et al. 2003, 2004; Vleugels and Van Bockstaele 2013; Hartmann et al. 2022). However, DNA markers reliably predicting resistance to both diseases would allow to substantially save time, effort, and resources through genomic prediction and early generation marker-assisted selection (MAS; Collard and Mackill 2008). Furthermore, MAS allows to combine multiple favored alleles through fewer crossing events when compared to pure phenotypic selection (Collard and Mackill 2008).

The main objective of this study was to better characterize disease resistance for the two most relevant fungal diseases threatening red clover production in Europe and other temperate zones worldwide and to identify genetic loci linked to resistance. Therefore, we screened a diverse collection of red clover accessions under controlled conditions for southern anthracnose and clover rot resistance. We examined the phenotypic variation in resistance to these two diseases, aiming to find accessions with a high degree of resistance to one or both diseases. Furthermore, we developed genome-wide allele frequency fingerprints using pooled genotyping-by sequencing (pool-GBS) and performed genome-wide association studies (GWAS) to identify quantitative trait loci (QTL). Potential candidate resistance genes were identified in the genomic regions underlying the QTL associated with southern anthracnose and clover rot resistance.

## Material and methods

We used a collection of 397 red clover accessions that was established in the frame of the EUCLEG project (Horizon 2020 Programme for Research & Innovation, grant agreement no. 727312; http://www.eucleg.eu). This collection (hereafter referred to as the EUCLEG-accessions) contains plant material from 23 countries including cultivars, breeding material, landraces, and ecotypes. Detailed information on the EUCLEG-accessions is given in Supplementary Table S1. Each EUCLEG-accession can be considered as a population of related plants. While all accessions were used for genotyping and phenotyping of southern anthracnose resistance, only 392 accessions were screened for clover rot resistance.

### Genotyping and filtering for single nucleotide polymorphisms (SNPs)

Seedlings were grown in the greenhouse in 96-compartment plant trays filled with compost. At the one-leaf stage, that leaf was harvested from 200 seedlings per accession. Fresh leaves from the same accession were pooled, and DNA was extracted using the QIAGEN DNeasy 96 Plant kit (QIAGEN, Citylabs 2.0, Manchester M13 0BH, UK). The DNA concentration was measured using a Qubit™2.0 instrument and normalized to 20 ng µl^−1^.

Genotyping was realized by LGC Genomics (Berlin, Germany) using a *Pst*I-*Mse*I double-digest pool-GBS method, in combination with PE-150 Illumina sequencing. Sequencing data covered 10,609 unique loci with an average read depth of 288. Average read length per locus was 188, resulting in a total sequence length of 2.0 Mb which corresponds to approximately 0.6% of the assembled draft genome sequence length (309 Mb; De Vega et al. 2015). SNP calling and allele frequency calculations were done as described in Keep et al. (2020). A detailed description of the parameters specific for this study is provided in the Supplementary Methods. Only biallelic SNPs were considered. Allele frequencies were called using SNAPE-pooled (Raineri et al. 2012). An allele frequency of one corresponds to homozygosity of the population for the reference allele, and a frequency of zero corresponds to homozygosity of the alternative allele. Data were filtered to retain SNPs with a minimum read depth of 30, less than 5% missing values, allele frequencies between 0.05 and 0.95 in at least 10 accessions, and mean allele frequencies across all accessions between 0.05 and 0.95 (0.05 < MAF < 0.95). After filtering, we obtained a total of 20, 137 SNPs. Missing data were replaced by the mean allele frequency across all accessions per SNP using a custom-made R script (available at 10.5281/zenodo.7034131). The GBS reads are available at NCBI under project number PRJNA842231.

### Experimental design and phenotyping

#### Southern anthracnose

A resolvable row–column design with two standard cultivars as controls (‘Pavo’ and ‘Milvus’) and four full replications was used. Experimental units consisted of 24 plants of the same accession sown together. Plants were grown in plastic boxes (300 × 400 × 145 mm) filled with cultivation substrate at a plant-to-plant distance of approx. 4 cm. One replicate consisted of 160 boxes, each box containing 72 plants of three different accessions (3 × 24 = 72). Spray inoculation was adapted from Schubiger et al. (2003). Briefly, plants were grown in a greenhouse (19–23 °C, 16-h light from sodium-vapor bulbs, > 100 μEm^−2^ s^−1^) at Agroscope (Zurich, Switzerland). After 6 weeks, plants were cut 3–4 cm above the ground and allowed to regrow for 2 weeks. The number of living plants per experimental unit (G) was determined before plants were inoculated with a single-spore isolate. Fungal spores of the isolate CTR 010103 (collected on red clover in 2001 in Ellighausen, Switzerland) were grown on potato dextrose agar (PDA) at around 18 °C in the dark and 12-h ultraviolet light per 24 h. After 10 days, spores were gently removed with sterile dH_2_O. The concentration of the spore suspension was adjusted with dH_2_O to 3.2–4.8 × 10^6^ spores ml^−1^ by counting spores under the microscope. For inoculation, approximately 40 ml spore suspension was used per box, wetting the plants from top to bottom using a spray gun compressor at 2 bar. The inoculated plants were covered with a polyethylene sheet for 5 days. Plants were cut four times at 14-, 42-, 70-, and 98-days post-inoculation (dpi). The survival rate (*S*_rate_) was assessed by counting the surviving plants (S) 2 weeks after the second cut (56 dpi) multiplied by 100 and divided by the total number of plants before inoculation (*G*; Eq. 1).

1 $$S_{{{\text{rate}}}} \left( \% \right) = \frac{100 \times S}{G}$$

After scoring, the surviving plants were re-inoculated with a mixture of seven additional *C. trifolii* single-spore isolates collected 2001 in Ellighausen (CTR010101, CTR010102, CTR010104, CTR010105, CTR010106, CTR010107, CTR010108). Inoculation was conducted as described above.

Seven weeks after the second inoculation (105 days after the first inoculation), the surviving plants were counted. Cumulative survival rate (CumS_rate_) was calculated as survivors after the second inoculation period (*S*_cum_) divided by *G* (Eq. 2).

2 $${\text{CumS}}_{{{\text{rate}}}} \left( \% \right) = \frac{{S_{{{\text{cum}}}} }}{G} \cdot 100\%$$

#### Clover rot

A total of 13 separate clover rot trials were performed, so that all accessions were screened in three full replicates. These trials comprised up to 52 trays containing 94 accessions with 36 plants each, along with two positive and two negative (non-inoculated) control trays (both fully sown with the control cultivar ‘Lemmon’). Plants were sown 8 weeks prior to inoculation in Quickpot® trays (HerkuPlast QP96T, InterGrow, Alter, Belgium) in peat substrate (Saniflor Beroepspotgrond, InterGrow, Aalter, Belgium). Each tray was seeded with three accessions: one in the three top rows (36 plants), one in the three bottom rows (36 plants), and the control cultivar ‘Lemmon’ in the two middle rows (24 plants). Plants were grown in the greenhouse (20–25 °C, 12-h light from TL lamps at 50 μEm^−2^ s^−1^) and watered when required. Three weeks prior to inoculation, plants were cut at 5 cm above the ground. A single-spore isolate derived from Cz.A 1 (Vleugels et al. 2013a) was chosen for further experiments, as it possessed the highest growth speed on PDA medium. Inoculum was prepared to contain approximately 8000 mycelium fragments ml^−1^ in sterile dH_2_O with 5 g l^−1^ glucose and 150 µl l^−1^ Tween 20 (Sigma-Aldrich, Germany). Two to 4 days prior to inoculation, trays were moved to a growth chamber (15 °C, 12 h light), where they were randomly placed on eight growing tables and watered until saturation. After inoculation, the tables were covered with caps made of transparent plastic foil and misted to increase humidity. Plants were sprayed with mycelium suspension until run-off, after which the plastic caps were closed, and the lights dimmed until the next morning. The negative control trays were sprayed with infection solution without inoculum. Water was misted over the plants at day 3 and day 6 dpi, and the plastic foil was replaced immediately after misting. After 9 days of incubation, the plastic foil was removed, and the disease incidence was scored on each plant using a scale from 1 (no symptoms) to 5 (completely dead plant). Subsequently, scores were converted into percentages through calculation of the resistance index (RI) adapted from Marum et al. (1994) as follows (Eq. 3).

3 $${\text{RI}} = \left| {\frac{{{\text{score}} - 5}}{4}{*}100{\text{\% }}} \right|$$

### Calculation of mean values per accession and heritabilities

Statistical analyses were carried out in R statistical software version 4.0.3 (R Core Team 2021) and RStudio version 1.3.1093 (RStudio Team 2020) and the mixed model package ASReml-R version 4.0 (Butler et al. 2017). Assumptions of homoscedasticity of variances and normality of residuals were met according to residual plots, except for the cumulative survival rate, which was thus square root transformed. Linear mixed model analyses for southern anthracnose resistance were performed using accessions and replicates as fixed effects and all other parameters as random effects in the model (Eq. 4).

4 $${\upgamma }_{hijk} = {\upmu } + {\uptau }_{h} + {\upgamma }_{k } + r_{ik} + c_{jk} + e_{hijk}$$

where *γ*_*hijk*_ is the survival rate or cumulative survival rate of the *h*-th accession in the *i*-th row and *j*-th column nested within *k*-th complete replicate, μ the general mean, $\tau_{h}$﻿ the effect of the *h*-th accession,* γ*_k_ the effect of *k*-th complete replicate, *r*_*ik*_ the effect of *i*-th row within *k*-th replicate, *c*_*jk*_ the effect of *j*-th column within *k*-th replicate, and *e*_*hijk*_ the residual error per experimental unit.

Linear mixed model analysis for clover rot resistance was performed using accessions as fixed effects and all other parameters as random effects in the model (Eq. 5).

5 $$\gamma_{hij} = \mu + \tau_{h} + r_{i} + c_{ij} + e_{hij}$$

where* γ*_*hij*_ is the resistance index of the *h*-th accession on the *j*-th table nested within the *i-*th trial, μ the general mean, $\tau$_*h*_ the effect of the *h*-th accession,* r*_*i*_ the effect of *i*-th trial,* c*_*ij*_ the effect of *j*-th table within *i*-th trial, and* e*_*hij*_ the residual error per experimental unit.

For fixed effects Wald *x*^*2*^ tests with ssType = “conditional” were performed. Best linear unbiased estimates (BLUEs) for each accession and all traits were calculated. Heritability was calculated according to Cullis et al. (2006; Eq. 6).

6 $$H_{{{\text{Cullis}}}}^{2} = 1 - \frac{{\overline{\vartheta }_{\Delta }^{{{\text{BLUP}}}} }}{{2*\delta_{g}^{2} }}$$

where* δ*^2^_*g*_ is the variance of the accession and ${\overline{\vartheta }}_{\Delta}^{\text{BLUP}}$ the average standard error of the accession BLUPs. Pairwise Wilcoxon rank sum tests were performed with a Bonferroni threshold of *α* = 5% to compare the different red clover accession types (i.e., ecotypes, landraces, breeding material, and cultivars). BLUEs were used as accession values for downstream analyses.

### Genomic relationship matrix and association between SNPs and phenotypic traits

The genomic relationship matrix was calculated as described in Cericola et al. (2018) with a ploidy number of 16, which is assumed to be ideal when dealing with synthetic cultivars. GWAS were performed with the multi-locus mixed-model (MLMM) approach implemented in the R package mlmm.gwas (Segura et al. 2012; Bonnafous et al. 2019). Through forward inclusion and backward elimination, SNPs were integrated as cofactors into a mixed-model regression approach. Variance components of the model were estimated at each step separately. The number of steps was limited to 20, and the model with the lowest Bayesian information criterion (BIC) was selected (Chen and Chen 2008). The effect sizes for the SNPs associated with resistance were given as the regression coefficient (*β*) derived from a linear mixed model with BLUEs corrected means as response variable, SNPs as fixed effects, and the kinship matrix as random effect. The percentage of phenotypic variation explained by each SNP was obtained by comparing the *R*^2^ of a linear model taking SNPs as fixed effects and the kinship matrix as random effect to the *R*^2^ of the same model without integrating the SNPs. Mixed linear models were calculated with the “lmekin” function of the “coxme” R package (Therneau 2020). SNP positions and adjacent regions of the genome annotation of the red clover reference genome sequence v2.1 (De Vega et al. 2015) were visualized using CLC genomic workbench version 9 (CLC bio, Aarhus, Denmark). Sequences of the genes containing the significant SNPs (Table 2) and genes in adjacent regions (10 kb up- and downstream) were compared to the *M. truncatula* genome (BLASTn; Tang et al. 2014), and the BLAST hit with the lowest *e*-value was selected.

## Results

**Fig. 1:**
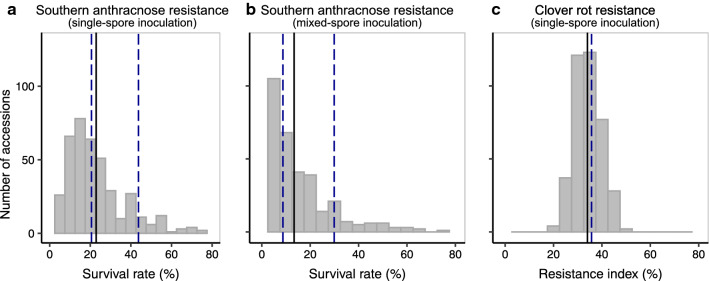
Frequency distribution of resistance scores of the red clover (*Trifolium pratense* L.) EUCLEG-accessions. Plots depict adjusted means for southern anthracnose survival rate (**a**), back-transformed cumulative survival rate (**b**), and resistance index for clover rot (**c**). Means are indicated by the solid line and compared with the control cultivars (dashed lines) ‘Milvus’ (left) and ‘Pavo’ (right) for southern anthracnose resistance (a and b), and ‘Lemmon’ for clover rot resistance (**c**)

Phenotypic variation among the 397 EUCLEG-accessions was high for southern anthracnose resistance. For the single-spore inoculation, the survival rate ranged from 0 to 79.9% (Fig. 1a), and for the mixed-spore inoculation the back-transformed cumulative survival rate (square root transformed) ranged from 0 to 73.5% (Fig. 1b). The overall mean was 22.9% for the single-spore inoculation and 13.1% for the mixed-spore inoculation. Mean survival rate of the rather susceptible cultivar ‘Milvus’ was 20.6% for the single-spore inoculation and 8.7% for the mixed-spore inoculation, which was only 2.3 and 4.4% lower than the overall mean of the trial for single-spore and mixed-spore inoculation, respectively (Fig. 1a, b). Mean survival rate of the cultivar ‘Pavo’ (43.7% for single-spore and 29.9% for mixed-spore inoculation) was 20.8 and 16.8% higher than the mean of all accessions for single-spore and mixed-spore inoculation, respectively.

For clover rot resistance, the phenotypic variation among the EUCLEG-accessions was smaller, with resistance indices ranging from 19.7 to 48.9% and a mean of 34.0% (Fig. 1c). The cultivar ‘Lemmon’ showed, with a resistance index of 35.7%, a similar resistance index as the average of all accessions.

Means and standard errors for the three traits and for all accessions are listed in Supplementary Table S2. Variance components for the three traits were significant for accession effects (Table 1). Heritability was high, with 0.85 for southern anthracnose resistance (single-spore and mixed-spore inoculation), and 0.89 for clover rot resistance. Comparable values for heritability were obtained with the method of Piepho and Möhring (2007; data not shown). The high heritabilities can be explained by the low average standard error of the accession BLUPs achieved by replicated artificially inoculated greenhouse trials.

**Table 1:** Mean survival rates for southern anthracnose and resistance index for clover rot as well as Cullis heritabilities (*H*^2^)

Trait	Survival rate/resistance index (%)	*H*^2^	df	*F*-value	*p-*value
Southern anthracnose (single-spore inoculation)	22.87	0.85	396	8.31	< 0.001
Southern anthracnose (mixed-spore inoculation^a^)	13.12^b^	0.85	396	7.83	< 0.001
Clover rot (single-spore inoculation)	34.04	0.89	392	9.95	< 0.001

^a^Square root transformed

^b^Back-transformed

**Fig. 2:**
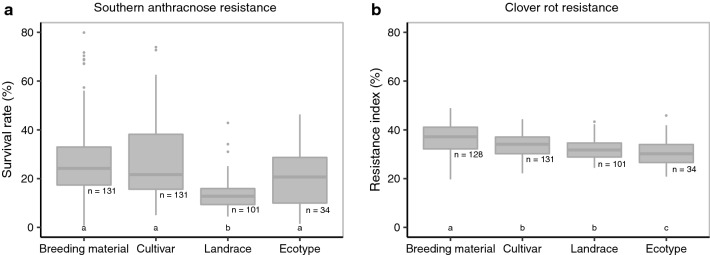
Accession means grouped according to the type of accession for survival rate after southern anthracnose single-spore inoculation (**a**) and resistance index for clover rot (**b**). Horizontal bars represent medians. Medians with no letter in common were significantly different (Kruskal–Wallis; *α* = 5%)

**Fig. 3:**
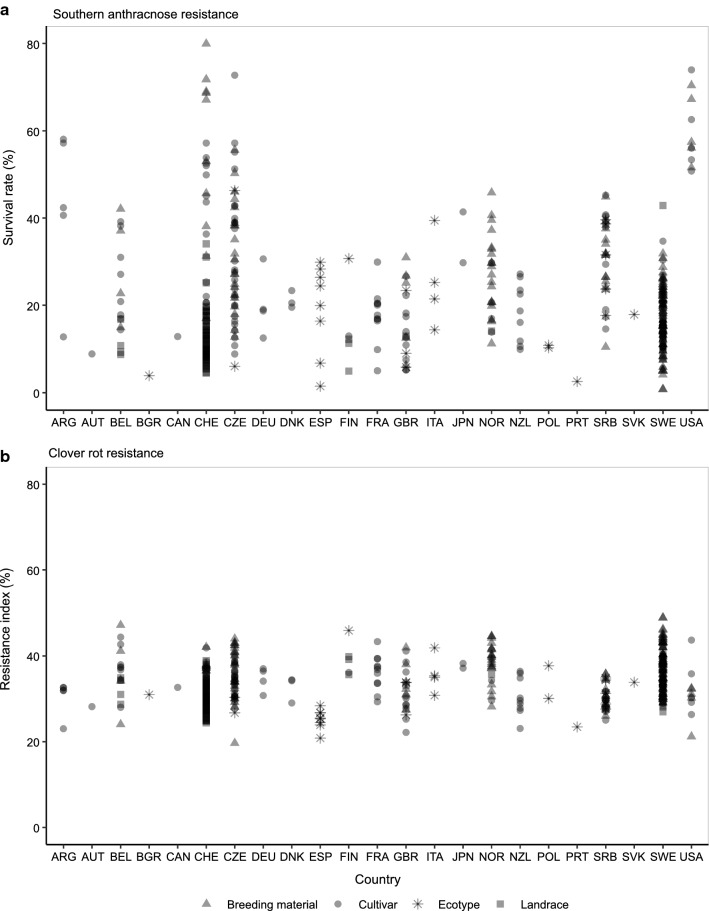
Accession means grouped according to their origin (three letter country code). Survival rates for single-spore inoculation (**a**) and resistance index for clover rot (**b**)

Grouping the accessions into breeding material, cultivars, landraces, and ecotypes revealed significant differences (*p* < 0.05) among the different groups (Fig. 2). For southern anthracnose, landraces showed a significantly lower survival rate compared to the other three types of material. A high variation within the four groups for single-spore inoculation was observed (Fig. 2a). The results for the mixed-spore inoculation were comparable (data not shown). For clover rot resistance, breeding material performed slightly, but significantly (*p* < 0.05), better than cultivars, landraces, and ecotypes. The variation in clover rot resistance within the four groups was low compared to southern anthracnose resistance and reflected the overall lower level of variation. Landraces and cultivars from the USA showed a generally high resistance to southern anthracnose with the lowest survival rate being as high as 50.8%. Substantial resistance was also observed in breeding material and cultivars from Argentina, Czech Republic, and Switzerland (Fig. 3a). For clover rot resistance, breeding material from Sweden and Norway performed best with resistance indices of 48.9 and 44.6%, respectively (Fig. 3b).

Genome-wide allele frequency fingerprints retained a total of 20,137 SNPs with 0.7% missing values after filtering. SNP reference allele frequencies across all accessions were biased toward one, indicating that many low-frequency alternative alleles exist, while the most abundant allele across the accessions is consistent with the nucleotide encoded in the reference genome sequence (left skewed; Supplementary Fig. S1). A total of 7372 SNPs were located on scaffolds with unassigned chromosomal position. The other 12,765 SNPs (63.4%) were evenly spread across the seven chromosomes. The average SNP density of the SNPs assigned to the seven chromosomes was 26.85 SNPs per 250 kb (Supplementary Fig. S2). Linkage disequilibrium (LD) of adjacent SNPs (Supplementary Fig. S3) was almost absent, and therefore, flanking genes were only identified in the 10 kb regions up- and downstream of significant SNPs.

**Fig. 4:**
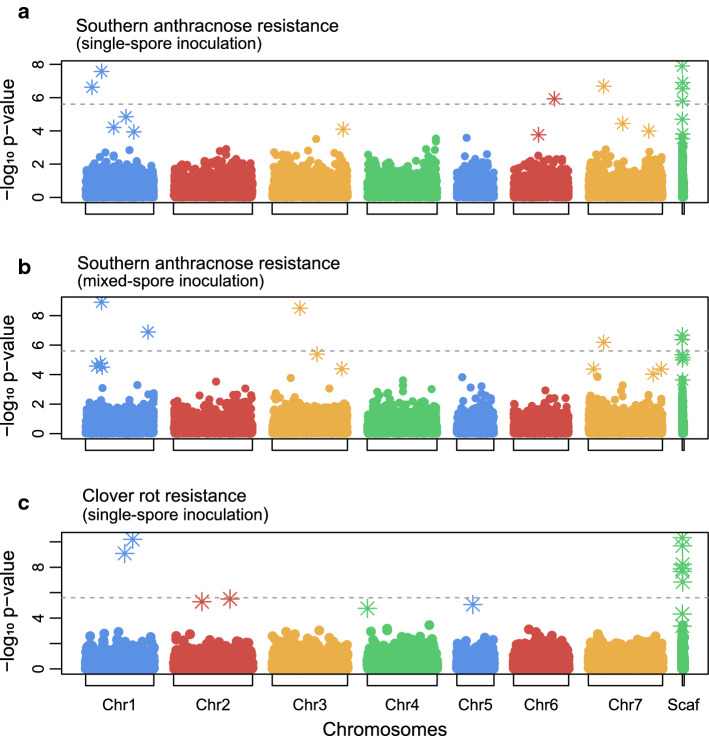
Genome-wide association study based on 20,137 single nucleotide polymorphisms using the multi-locus mixed-model approach (Segura et al. 2012), for survival rate after southern anthracnose single-spore inoculation (**a**), mixed-spore inoculation (**b**), and resistance index for clover rot (**c**). The dotted line represents the significance threshold after Bonferroni correction (*α* = 5%)

We found several SNPs that were significantly associated with each of the three traits investigated (Fig. 4). The SNP “LG1_6601280” explained 16.8% (*β* =−89.4) and 14.3% (*β* =−85.1) of the total phenotypic variation for southern anthracnose resistance after single-spore inoculation and mixed-spore inoculation, respectively. For clover rot resistance the most relevant SNP “Scaf658_16191” explained 12.8% (*β* = 13.8) of the total phenotypic variation. Of another five SNPs, each explaining about 10% of the phenotypic variation for clover rot resistance, two were located on chromosome 1. For all traits combined, a total of 22 SNPs were significantly associated after Bonferroni correction (α = 5%), of which 18 were found in coding regions of the reference genome. The function of these genes in red clover was assigned based on orthology to *M. truncatula* genes (Table 2).

**Table 2:** Single-nucleotide polymorphisms associated with southern anthracnose and clover rot resistance

SNP ID	Chr	Position (bp)	Coefficient(*β*)	Variance explained (%)	Red clover gene containing SNP	Annotation in *Medicago truncatula*
Southern anthracnose resistance, single-spore inoculation						
LG1_6601280	1	6,601,280	− 89.4^a^	16.8	*Tp57577_TGAC_v2_gene4880*	*MTR_1g103020* 3-oxoacyl- (acyl carrier) synthase II
LG7_6328288	7	63,282,88	− 56.3^a^	9.0	*Tp57577_TGAC_v2_gene32177*	*MTR_3g117330* kinesin motor catalytic domain protein
LG6_16830298	6	16,830,298	25.1^a^	6.7	*Tp57577_TGAC_v2_gene23646*	*MTR_2g049640* RNA recognition motif 2 in plant MEI2-like protein
Scaf595_126629	Scaf595	126,629	− 20.9^a^	6.3	No gene	NA
LG1_2684496	1	2,684,496	− 30.0^a^	4.4	*Tp57577_TGAC_v2_gene14494*	*MTR_1g054675* RNA recognition motif
Scaf323_195382	Scaf 323	195,382	19.4^a^	4.0	*Tp57577_TGAC_v2_gene19649*	*MTR_1g041890* peptide chain release factor, putative
Scaf215_144740	Scaf 215	144,740	− 20.0^a^	3.6	*Tp57577_TGAC_v2_gene10592*	*MTR_2g026595* Hypothetical protein
Scaf8613_1314	Scaf 8613	1,314	− 36.8^a^	3.5	*Tp57577_TGAC_v2_gene22635*	*MTR_3g010210* nucleic acid-binding, OB-fold-like protein
Southern anthracnose resistance, mixed-spore inoculation						
LG1_6601280	1	6,601,280	− 85.^bd^	14.3	*Tp57577_TGAC_v2_gene4880*	*MTR_1g103020* 3-oxoacyl-(acyl carrier) synthase II
LG1_25782873	1	25,782,873	19.7^bd^	6.3	No gene	NA
Scaf753_87981	Scaf 753	87,981	19.9^bd^	3.9	*Tp57577_TGAC_v2_gene29929*	Nothing found
Scaf10964_710	Scaf 10,964	710	18.7^bd^	2.8	*Tp57577_TGAC_v2_gene39892*	*MTR_5g042410* acylamino-acid-releasing enzyme-like protein, putative
LG3_11481441	3	11,481,441	44.0^bd^	2.6	*Tp57577_TGAC_v2_gene11903*	*MTR_3g037570* peptidylprolyl cis/trans isomerase, NIMA-interacting protein
LG7_6328288	7	6,328,288	− 34.7 ^bd^	2.1	*Tp57577_TGAC_v2_gene32177*	*MTR_3g117330* kinesin motor catalytic domain protein
Clover rot resistance, single-spore inoculation						
Scaf658_16191	Scaf 658	16,191	13.8^c^	12.8	*Tp57577_TGAC_v2_gene35789*	*MTR_7g079030* proline iminopeptidase-like protein
LG1_19399829	1	19,399,829	13.1^c^	12.4	*Tp57577_TGAC_v2_gene37747*	*MTR_1g090690* putative disease resistance RPP13-like protein 3
Scaf509_74106	Scaf 509	74,106	10.4^c^	11.2	*Tp57577_TGAC_v2_gene10001*	*MTR_6g015285* nuclear matrix constituent-like protein
Scaf1864_1986	Scaf1864	1986	−7.8^c^	10.5	*Tp57577_TGAC_v2_gene5179*	*MTR_4g058730* cyclic nucleotide-gated ion channel protein
LG1_16117955	1	16,117,955	−18.0^c^	9.5	*Tp57577_TGAC_v2_gene33326*	*MTR_1g029410* small RNA degrading nuclease
Scaf430_104086	Scaf 430	104,086	7.8^c^	9.4	No gene	Na
Scaf512_143846	Scaf 512	143,846	14.8^c^	8.5	No gene	Na
Scaf3654_3161	Scaf 3654	3161	−6.4^c^	5.9	*Tp57577_TGAC_v2_gene968*	*MTR_3g027470* putative disease resistance RPP13-like protein 1

SNP ID, chromosome (Chr), SNP position on the chromosome, effect size as regression coefficient (*β*), phenotypic variance explained by that SNP, red clover gene containing the SNP, and the gene name and gene function of the closest ortholog in *M. truncatula* are listed

^a^Intercept = 82.4

^b^Intercept = 48.5

^c^Intercept = 41.0

^d^Back-transformed BLUEs corrected means

## Discussion

Most of the 397 red clover accessions showed a high susceptibility to southern anthracnose and clover rot. In recent decades, summers in Central Europe became warmer and *C. trifolii* infections increased (Boller et al. 2010). Winters became damper and long periods of dry frost became less likely, conditions which favor clover rot infections (Öhberg 2008). Southern anthracnose and clover rot became a limiting factor for red clover production in regions that were previously not affected (Boller et al. 2010; Jacob et al. 2015). Changing climatic conditions can substantially shape crop pathogen assemblages (Chaloner et al. 2021). The lack of adaptation to the newly emerging pathogens may explain the overall high susceptibility for southern anthracnose and clover rot observed in the red clover EUCLEG-accessions.

In general, ecotypes from Southern European countries showed a higher survival rate after inoculation with *C. trifolii* (Fig. 3a), compared to populations from Northern latitudes. Also, US cultivars and breeding materials performed well with a high survival rate after spray inoculation with *C. trifolii*. This may be explained by the intense selection efforts for southern anthracnose resistance in the USA since the 1950s in Southern regions (Taylor 2008). For cultivars coming from the Northern USA, where selection for southern anthracnose was not a main selection target, the high resistance might also be explained by a direct response of natural selection against the pathogen (Taylor 1985). Landraces, ecotypes, cultivars, and breeding material from Sweden and Finland showed a higher resistance index for clover rot compared to populations from the other regions (Fig. 3). In Sweden, clover rot is the major cause of red clover stand failure since decades and early generation selection for clover rot resistance is indispensable (Lundin and Jönsson 1974). Some accessions from Belgium, Switzerland, and the Czech Republic, mainly new breeding material, showed an increased resistance to either one or both diseases (Fig. 3), reflecting more recent attempts to actively select for southern anthracnose and clover rot resistance. These observations are consistent with the hypothesis that resistance levels increase in regions where pathogens occur, by adaptation through natural or artificial selection over time (Huxley 1939; Burdon and Thrall 2009). Both types of selection have probably played a role in shaping the geographical differentiation in levels of red clover disease resistance against the pathogens investigated in this study.

Despite the generally high susceptibility of the EUCLEG-accessions, there was considerable phenotypic variation for resistance.

We observed a high variation in resistance to southern anthracnose, whereas the phenotypic variation for clover rot resistance was comparably low. For southern anthracnose, several accessions did show a high survival rate and could present a valuable resistance source for breeding programs. On the other hand, no accession showed an appropriate level for clover rot resistance, thus hampering direct introgression of resistance into existing breeding material. However, recurrent selection after artificial inoculation has previously been shown to improve levels of resistance against clover rot in red clover and resistance to *S. sclerotiorum* in different legume species (Terán and Singh 2009; Vleugels et al. 2013b). Recurrent selection after artificial inoculation trials seems to date the only option to substantially increase resistance levels for clover rot as well as for southern anthracnose in red clover (Schubiger et al. 2003; Vleugels et al. 2013b; Jacob et al. 2015).

Despite considerable success, phenotypic recurrent selection using artificial spray inoculation is time and labor intensive and requires large greenhouse trials. Furthermore, the fixation of resistance alleles in population-based cultivars is difficult (Patella et al. 2019). DNA markers that are linked to specific QTL harboring genes with a specific role in resistance, so-called diagnostic markers, are routinely used in cultivar development of major crops like wheat (*Triticum aestivum* L.) and maize (*Zea Mays* L.; Miedaner and Flath 2007; Guo et al. 2019). MAS is particularly effective for qualitative resistance traits where only a few genes underlying the resistance are involved (Adam-Blondon et al. 1994; Xiao et al. 2001; Zhou et al. 2001).

Current breeding programs would benefit if selection for resistant cultivars could be realized using genetic markers. For southern anthracnose we found one locus on chromosome 1 explaining more than 16.8% of the variation in resistance to single-spore inoculation and 14.3% of the variation in resistance to the mixed-spore inoculation. Effect size (regression coefficient *β*) for the SNP “LG1_6601280” was −89.4 for the single-spore isolate and − 85.1 for the mixed-spore isolate (Table 2 and Supplementary Fig. S4).

While the genetic basis of southern anthracnose resistance in red clover is largely unknown, resistance to *Colletotrichum* spp. has been studied in other legume species including soybean, common bean, and the model species *M. truncatula* (Ameline-Torregrosa et al. 2008; Yang et al. 2008).

In alfalfa resistance to southern anthracnose is characterized by a strong hypersensitive response, typical for effector-triggered immunity or race-specific resistance (Mould et al. 1991). Consequently, three different *C. trifolii* races (1, 2, 4) and two resistance genes (*An1*, *An2*) have been described (Elgin and O’Neill 1988; O’Neill 1989; Mould et al. 1991). Elgin and Ostazeski (1985) stated that resistance to race 1 in the tetraploid cultivar ‘Arc’ is induced by a single-dominant gene *An1* that is tetrasomically inherited. *An2* conferred resistance to races 1 and 2 in the cultivar ‘Saranac AR.’ It is generally accepted that the two genes act independently and are not linked, but the effect of *An1* can be masked by the presence of *An2* (Elgin and Ostazeski 1985; O’Neill 1989). Unfortunately, the model of single tetrasomic gene inheritance could not be verified using Australian germplasm. Depending on the plant material, resistance was either simply inherited and of qualitative nature or of quantitative nature where several QTL with small-to-medium effects were involved in resistance (Mackie et al. 2003, 2007; Irwin et al. 2006).

In *M. truncatula*, where extensive genomic and genetic resources are available, a major QTL on linkage group (LG) 4 governed resistance to *C. trifolii* race 1 and race 2, while a minor QTL on LG6 was only found when inoculated with *C. trifolii* race 1. The QTL on LG4 explained about 40% of the total phenotypic variation and contained a cluster of *NLR* genes (Ameline-Torregrosa et al. 2008). A single-dominant gene named *RCT1* on chromosome 4 was mapped in an F2 population that conferred resistance to race 1 (Yang et al. 2008). The *RCT1* gene of *M. truncatula* was transferred into susceptible alfalfa plants. The alfalfa plants carrying the *RCT1* gene from *M. truncatula* were resistant to all three *C. trifolii* races (Yang et al. 2008). Orthologs of *RCT1* were identified in the red clover reference genome sequence using BLASTn, but these genes were not in the flanking regions of any SNPs significantly associated with southern anthracnose resistance in the red clover EUCLEG-accessions. Nevertheless, the significantly associated SNPs and their 10-kb flanking regions did contain orthologs of putative resistance genes of *M. truncatula*. One SNP explaining 16.8% of the variation to southern anthracnose resistance is located in the red clover gene *Tp57577_TGAC_v2_gene4880*. Its *M. truncatula* ortholog *MTR_1g10302* plays a role in fatty acid synthesis, and its *A. thaliana* ortholog, known as *KASI*, is involved in lipid metabolism and plays a role in cell structure and several plant developmental processes (Wu and Xue 2010). Pathways controlling fatty acid metabolism can play significant roles in cuticular plant defense (Kachroo and Kachroo 2009); hence, we speculate that the gene *Tp57577_TGAC_v2_gene4880* might be involved in resistance to southern anthracnose.

We found eight SNPs that were significantly associated with clover rot resistance, explaining together 80.2% of the total phenotypic variation (Table 2). Our study is the first to report loci associated with *S. trifoliorum* resistance in red clover. QTL for resistance to the related species *S. sclerotiorum* have been identified in legume crops such as soybean (Kim and Diers 2000; Arahana et al. 2001) and common bean (Park et al. 2001; Miklas 2007). Most of these QTL explained between 10 and 23% of the variation, which is comparable to the QTL identified in our study. The significant SNPs found for clover rot resistance are located in genes on chromosomes 1 and in scaffolds not assigned to chromosomes. The orthologs of the gene *Tp57577_TGAC_v2_gene37747* and the gene *Tp57577_TGAC_v2_gene968* are known in *M. truncatula* to be putative resistance genes. These putative disease resistance genes encode the RPP13-like protein, which in *Arabidopsis* has been reported to be involved in protection of plants against pathogen invasion by triggering a specific defense system against downy mildew (Bittner-Eddy et al. 2000). Resistance to clover rot in red clover is widely assumed to be a quantitative trait (Poland et al. 2009; Klimenko et al. 2010; Vleugels and Van Bockstaele 2013). For quantitative resistance traits, MAS is often ineffective due to population-specific effects and the lack of validation in unrelated populations (Miedaner and Korzun 2012). Therefore, MAS is most likely not effective enough to successfully replace artificial inoculation to select for clover rot resistance. Genomic selection might be a more promising approach (Miedaner et al. 2020). Based on literature (Mackie et al. 2007; Yang et al. 2008) and since only one SNP explained a substantial portion of the variation, we assume that inheritance of southern anthracnose resistance in red clover is mono- or oligogenic. If the effect of the QTL found here can be confirmed, implementing MAS in red clover breeding programs will become a feasible strategy to improve southern anthracnose resistance.

We conducted association studies on allele frequencies per population, allowing to reveal the high genetic population variation of outcrossing species such as red clover, without the need to sequence thousands of individuals (Byrne et al. 2013). If sequencing resources are limited, association studies on population level provide a valuable method and have been effective in finding important loci in humans (Riaz et al. 2016) and to a lesser extent also in plants (Cericola et al. 2018; Keep et al. 2020). Nevertheless, to validate the significant loci and to further characterize resistance alleles, genotyping of single plants is necessary.

Given current and predicted future disease pressure, cultivars that are resistant to southern anthracnose and to clover rot are urgently needed to ensure successful red clover production in Central Europe. Independent inheritance seems likely since Pearson correlation between accession means for the two diseases was absent (0.058; data not shown), and resistance loci found in this study were on different chromosomes (scaffolds) or far apart and are most likely not linked. Therefore, the chances are high that the two diseases are not associated and combining them in one population seems reasonable. We expect that our findings provide a path forward to increase efficiency in breeding for disease resistance in red clover.

## Supplementary Information

Below is the link to the electronic supplementary material.

Supplementary file1 (PDF 1869 kb)

Supplementary file2 (PDF 269 kb)

Supplementary file3 (PDF 368 kb)

Supplementary file4 (PDF 343 kb)

## Data Availability

Raw data and scripts can be accessed via: 10.5281/zenodo.7034131. GBS reads are available at NCBI under project number PRJNA842231.

## Abbreviations

GBS: Genotyping-by-sequencing
GWAS: Genome-wide association studies
MAS: Marker-assisted selection
