# Supplementary material for: Phenotypic variation and quantitative trait loci for resistance to southern anthracnose and clover rot in red clover
Source: Theor Appl Genet. 2022 Sep 25;135(12):4337–49. doi: 10.1007/s00122-022-04223-8 (PMC9734235; doi:10.1007/s00122-022-04223-8)
Supplement: Supplementary file 1 — Supplementary file1 (PDF 1869 kb) [file 122_2022_4223_MOESM1_ESM.pdf]

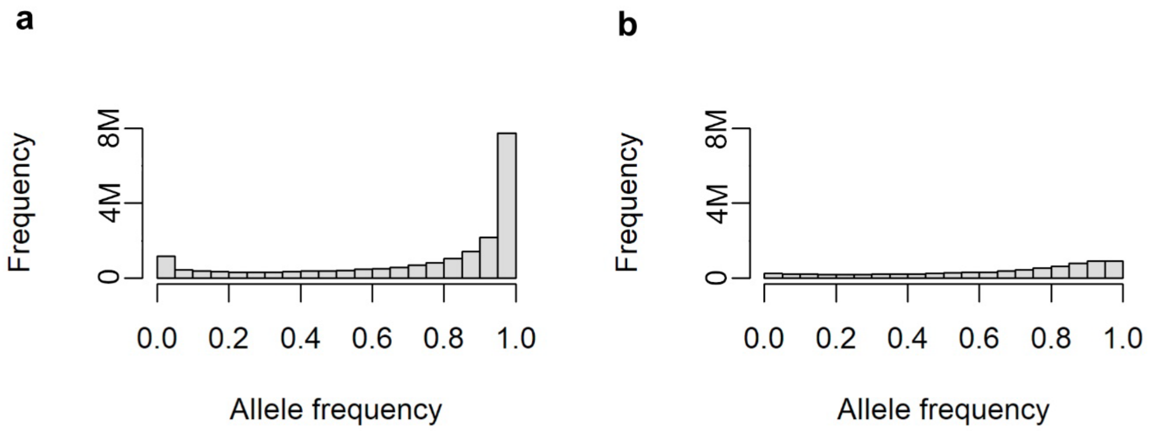

Supplementary Fig. S1 Allele frequency distribution of all 20,137 single nucleotide polymorphisms (SNPs) before (a) and after (b) filtering. Filtering was performed retaining SNPs with less than 5% missing values, allele frequencies between 0.05 and 0.95 in at least 10 accessions and mean allele frequencies across all accessions between 0.05 and 0.95 (0.05 < MAF < 0.95)

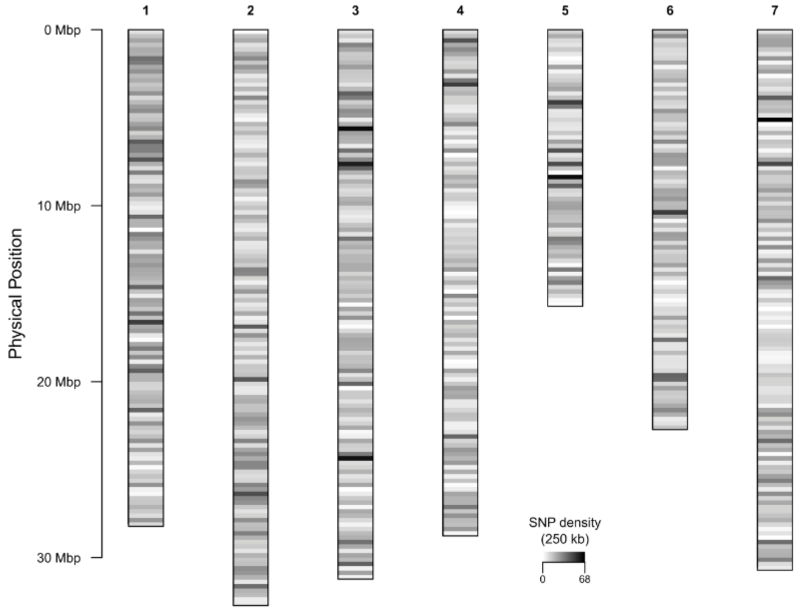

Supplementary Fig. S2 Single nucleotide polymorphism (SNP) density on the seven *Trifolium pratense* chromosomes. The y-axis represented the interval distance in Mbp. The window size to calculate SNP density was 250kb

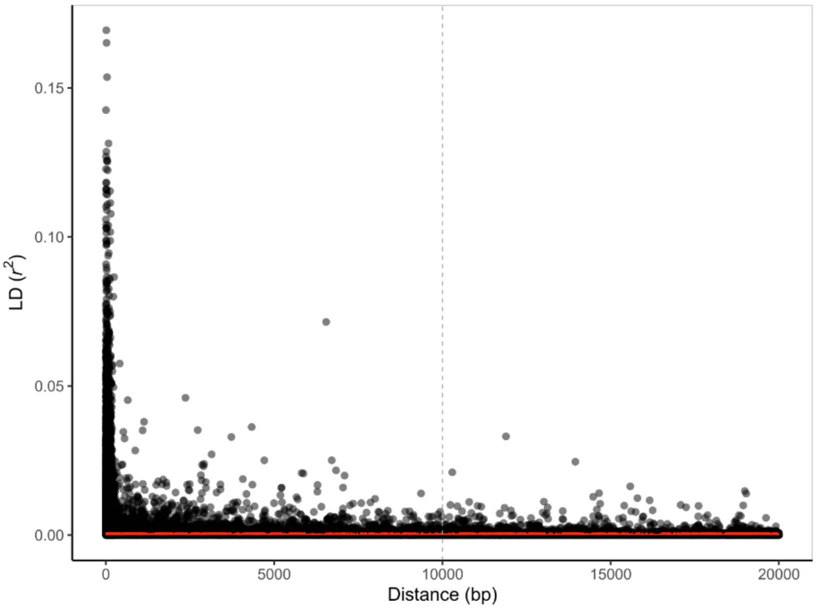

Supplementary Fig. S3 Linkage disequilibrium (LD) decay against the genetic distance for pairs of SNPs across all seven chromosomes of the red clover genome. LD estimates are reported as squared correlations of allele frequencies (r^2). The red solid line depicts the smoothed fitted line calculated using the LOESS method. Vertical dotted line is drawn at 10,000 bp

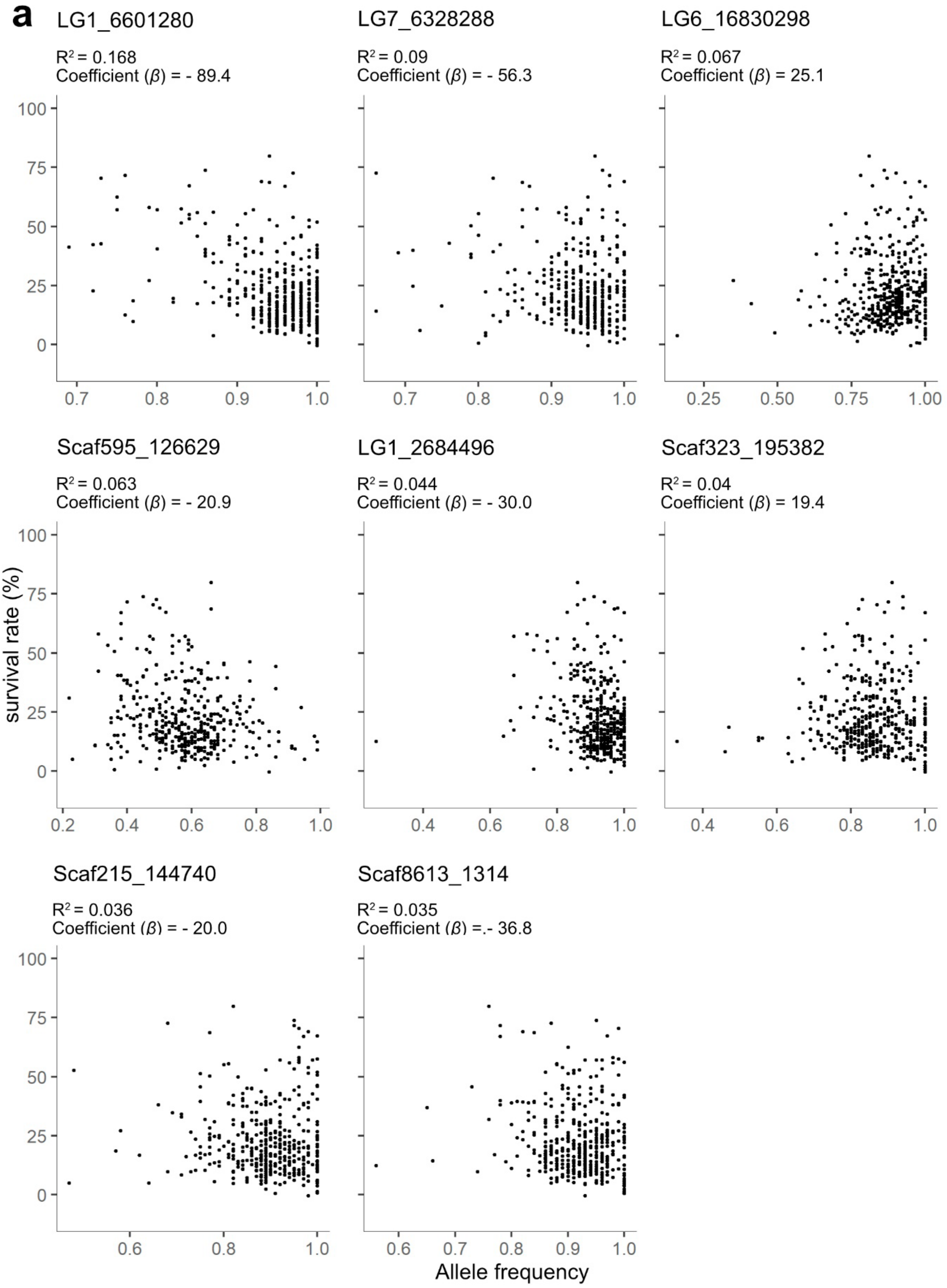
a
LG1_6601280
R^2 = 0.168
Coefficient (β) = - 89.4
LG7_6328288
R^2 = 0.09
Coefficient (β) = - 56.3
LG6_16830298
R^2 = 0.067
Coefficient (β) = 25.1
Scaf595_126629
R^2 = 0.063
Coefficient (β) = - 20.9
LG1_2684496
R^2 = 0.044
Coefficient (β) = - 30.0
Scaf323_195382
R^2 = 0.04
Coefficient (β) = 19.4
Scaf215_144740
R^2 = 0.036
Coefficient (β) = - 20.0
Scaf8613_1314
R^2 = 0.035
Coefficient (β) =.- 36.8
survival rate (%)
Allele frequency

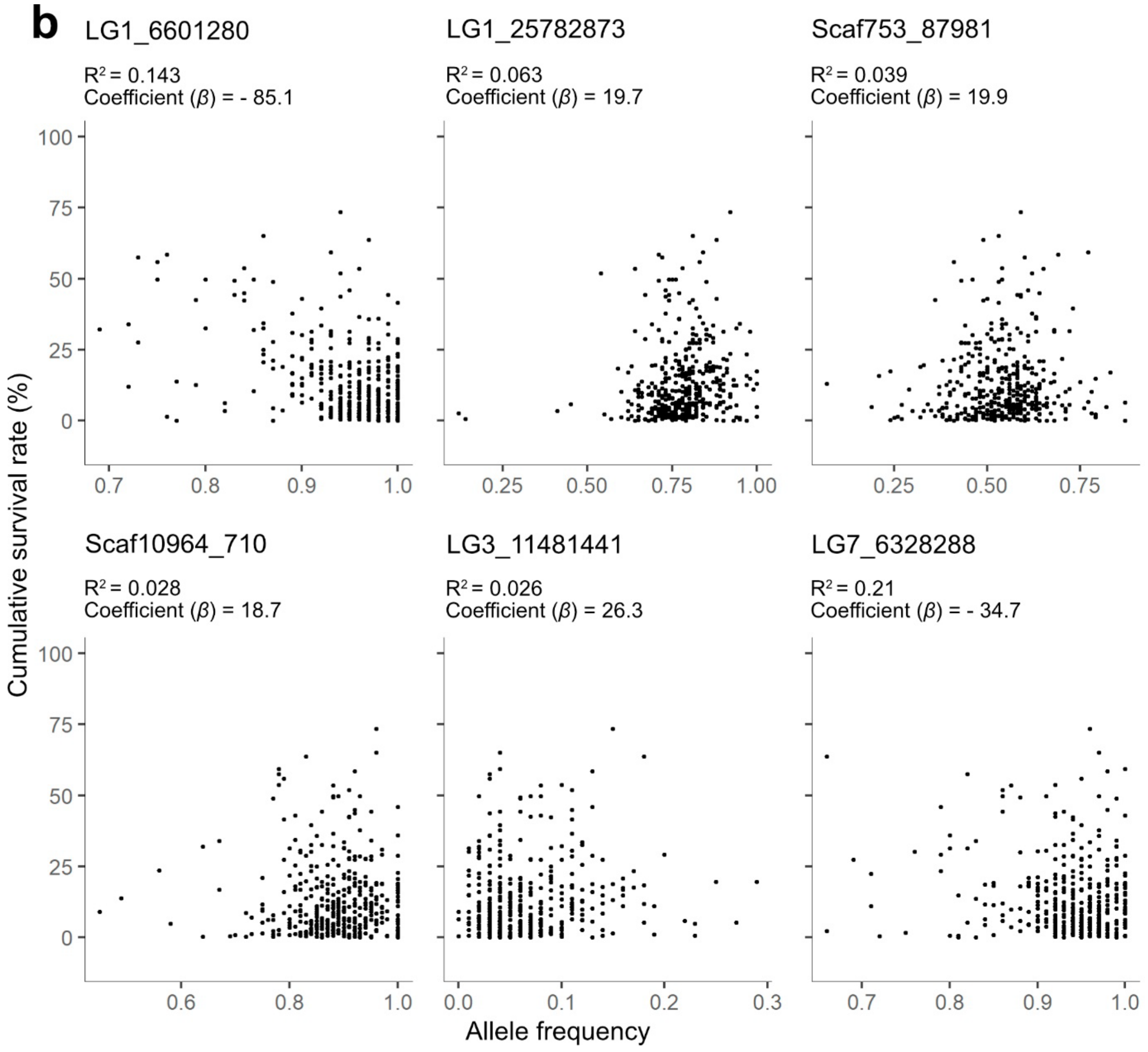
b
LG1_6601280
R² = 0.143
Coefficient (β) = - 85.1
LG1_25782873
R² = 0.063
Coefficient (β) = 19.7
Scaf753_87981
R² = 0.039
Coefficient (β) = 19.9
Scaf10964_710
R² = 0.028
Coefficient (β) = 18.7
LG3_11481441
R² = 0.026
Coefficient (β) = 26.3
LG7_6328288
R² = 0.21
Coefficient (β) = - 34.7
Cumulative survival rate (%)
Allele frequency

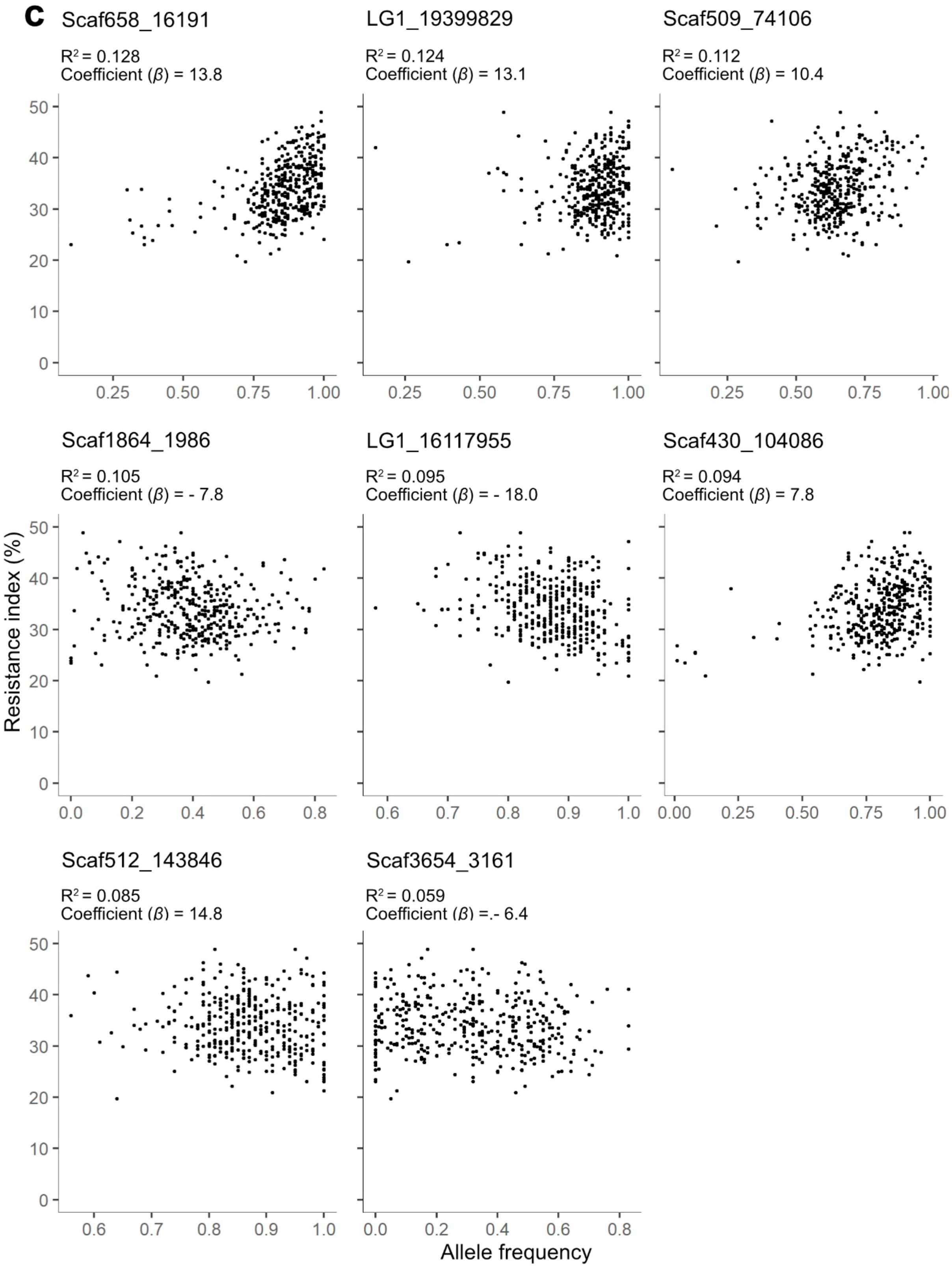

Supplementary Fig. S4 Scatter plots of the BLUEs corrected mean phenotypic observations and allele frequencies separate for each significantly associated SNP. Horizontal axes show the allele frequencies. Vertical axes depict the survival rate (%) for single-spore inoculation (a), back-transformed cumulative survival rate (%) for mixed-spore inoculation (b), and resistance index for

clover rot (c). The coefficient of determination R^2 is calculated by comparing a linear model taking SNPs as fixed effects and the kinship matrix as random effect to the R^2 of the same model without integrating the SNPs. Coefficient (β) is the slope of a linear model taking phenotypic observations as response variable, significantly associated SNPs as fixed effects and the kinship matrix as random effect
