# Supplementary material for: Phenotypic variation and quantitative trait loci for resistance to southern anthracnose and clover rot in red clover
Source: Theor Appl Genet. 2022 Sep 25;135(12):4337–49. doi: 10.1007/s00122-022-04223-8 (PMC9734235; doi:10.1007/s00122-022-04223-8)
Supplement: Supplementary file 3 — Supplementary file3 (PDF 368 kb) [file 122_2022_4223_MOESM3_ESM.pdf]

Supplementary Table S1 EUCLEG ID, Name of accession, country of origin, year of cultivar registration or collection, type of material as well as collection sites for ecotypes for all accessions used in this study

EUCLEG ID	Name of accession	Provenance country / Country of origin	Year of cultivar registration / year of collection	Type	Collection site Latitude	Longitude
EUC_TP_001	Dimanche	FRA	2006	Cultivar		
EUC_TP_002	Discovery	FRA	2006	Cultivar		
EUC_TP_003	Formica	CHE	1993	Cultivar		
EUC_TP_004	Milvus	CHE	1993	Cultivar		
EUC_TP_005	Pavo	CHE	2002	Cultivar		
EUC_TP_006	S586 AberClaret	GBR	2005	Cultivar		
EUC_TP_007	S592 AberChianti	GBR	2006	Cultivar		
EUC_TP_008	Gandalf	NOR	2015	Cultivar		
EUC_TP_009	Lea	NOR	2002	Cultivar		
EUC_TP_010	K 17	SRB	NA	Cultivar		
EUC_TP_011	K 38	SRB	NA	Cultivar		
EUC_TP_012	SW Ares	SWE	2001	Cultivar		
EUC_TP_013	Cyllene	CZE	2015	Cultivar		
EUC_TP_014	Himalia	CZE	2012	Cultivar		
EUC_TP_015	Metis	CZE	2016	Cultivar		
EUC_TP_016	NA	FRA	2014	Cultivar		
EUC_TP_017	Saija	FIN	2006	Cultivar		
EUC_TP_018	Global	BEL	2002	Cultivar		
EUC_TP_019	Merviot	BEL	1980	Cultivar		
EUC_TP_020	Bonus	CZE	2008	Cultivar		
EUC_TP_021	NGB1132	FIN	1981	Landrace		
EUC_TP_022	NGB1133	FIN	1981	Landrace		
EUC_TP_023	NGB1142	FIN	1981	Landrace		
EUC_TP_024	NGB14322	FIN	1998	Ecotype	NA	NA
EUC_TP_025	NGB1730	DNK	1964	Cultivar		
EUC_TP_026	NGB2161	NOR	NA	Landrace		
EUC_TP_027	NGB2391	SWE	NA	Landrace		
EUC_TP_028	NGB2392	SWE	NA	Landrace		
EUC_TP_029	NGB2458	SWE	NA	Landrace		

EUC_TP_030	NGB2461	SWE	NA	Landrace
EUC_TP_031	NGB2487	SWE	NA	Landrace
EUC_TP_032	NGB2490	SWE	NA	Landrace
EUC_TP_033	NGB2492	SWE	NA	Landrace
EUC_TP_034	NGB4089	SWE	NA	Landrace
EUC_TP_035	Grasslands Colenso	NZL	1989	Cultivar
EUC_TP_036	Sensation	NZL	2002	Cultivar
EUC_TP_037	Affoltern i.E._328	CHE	1972	Landrace
EUC_TP_038	Affoltern i.E._6	CHE	1972	Landrace
EUC_TP_039	Belpberg_225	CHE	1972	Landrace
EUC_TP_040	Belpberg_226	CHE	1972	Landrace
EUC_TP_041	Belpberg_229	CHE	1972	Landrace
EUC_TP_042	Bern_78	CHE	1972	Landrace
EUC_TP_043	Bowil_119	CHE	1972	Landrace
EUC_TP_044	Bruetten_3	CHE	1972	Landrace
EUC_TP_045	Bubikon_8	CHE	1972	Landrace
EUC_TP_046	Burgistein_300	CHE	1972	Landrace
EUC_TP_047	Columba	CHE	2016	Cultivar
EUC_TP_048	Corvus	CHE	1995	Cultivar
EUC_TP_049	Dafila	CHE	2008	Cultivar
EUC_TP_050	Frauenkappelen_86	CHE	1972	Landrace
EUC_TP_051	Goldbach i.E._167	CHE	1972	Landrace
EUC_TP_052	Huttwil_50	CHE	1972	Landrace
EUC_TP_053	Huttwil_60	CHE	1972	Landrace
EUC_TP_054	Koeniz_231	CHE	1972	Landrace
EUC_TP_055	Koeniz_279	CHE	1972	Landrace
EUC_TP_056	Krauchthal_176	CHE	1972	Landrace
EUC_TP_057	Lanzenhaeusern_291	CHE	1972	Landrace
EUC_TP_058	Lestris	CHE	2009	Cultivar
EUC_TP_059	Merula	CHE	2002	Cultivar
EUC_TP_060	Milonia	CHE	2006	Cultivar
EUC_TP_061	Monaco	CHE	2012	Cultivar
EUC_TP_062	Niederwangen_262	CHE	1972	Landrace
EUC_TP_063	Niederwangen_75	CHE	1972	Landrace
EUC_TP_064	Oberthal_121	CHE	1972	Landrace

EUC_TP_065	Oberuzwil_280	CHE	1972	Landrace		
EUC_TP_066	Pastor	CHE	2010	Cultivar		
EUC_TP_067	Riedbach_88	CHE	1972	Landrace		
EUC_TP_068	Rueegsau_160	CHE	1972	Landrace		
EUC_TP_069	Rueti_314	CHE	1972	Landrace		
EUC_TP_070	Schmidigen_336	CHE	1972	Landrace		
EUC_TP_071	Semperina	CHE	2016	Cultivar		
EUC_TP_072	Signau_140	CHE	1972	Landrace		
EUC_TP_073	Sumiswald_189	CHE	1972	Landrace		
EUC_TP_074	Ueberstorf_294	CHE	1972	Landrace		
EUC_TP_075	Ueberstorf_346	CHE	1972	Landrace		
EUC_TP_076	Ufhusen_52	CHE	1972	Landrace		
EUC_TP_077	Uttigen_2	CHE	1972	Landrace		
EUC_TP_078	Wynigen_335	CHE	1972	Landrace		
EUC_TP_079	Zaeziwil_125	CHE	1972	Landrace		
EUC_TP_080	Zaeziwil_127	CHE	1972	Landrace		
EUC_TP_081	Aa 3100	GBR	1948	Cultivar		
EUC_TP_082	Aa 3148	GBR	1948	Cultivar		
EUC_TP_083	Aa 3149	GBR	1948	Cultivar		
EUC_TP_084	AA 32	GBR	2011	Breeding material		
EUC_TP_085	Aa 3459	GBR	1957	Cultivar		
EUC_TP_086	Aa 4190	POL	1990	Ecotype	50.85	20.65
EUC_TP_087	Aa 4292	CZE	1992	Ecotype	49.483	18.1
EUC_TP_088	Aa 4298	SVK	1992	Ecotype	49.183	18.2167
EUC_TP_089	Aa 4351	BGR	1993	Ecotype	42.85	25.5667
EUC_TP_090	Aa 4379 Britta	GBR	1994	Cultivar		
EUC_TP_091	Aa 4390	PRT	1995	Ecotype	41.333	-7.7667
EUC_TP_092	Aa 4400	GBR	1996	Ecotype	54.65	-2.15
EUC_TP_093	Aa 4444	ITA	1998	Ecotype	46.017	12.4833
EUC_TP_094	Aa 4516	ESP	2003	Ecotype	43.305	-4.8352
EUC_TP_095	Aa 4519	ESP	2003	Ecotype	42.913	-5.9247
EUC_TP_097	Aa 4528	ESP	2003	Ecotype	43.412	-5.3078
EUC_TP_098	Aa 4939	NZL	2003	Cultivar		
EUC_TP_099	Aa 5674	ARG	2016	Cultivar		
EUC_TP_100	Aa 5675	ARG	2016	Cultivar		

EUC_TP_101	Aa 5676	ARG	2016	Cultivar
EUC_TP_102	Aa 5677	ARG	2016	Cultivar
EUC_TP_103	Aa 5678	ARG	2016	Cultivar
EUC_TP_104	Aa 5746 Harmonie	GBR	2017	Cultivar
EUC_TP_105	Aa4380 Altaswede	CAN	NA	Cultivar
EUC_TP_106	S543 AberRuby	GBR	2000	Cultivar
EUC_TP_108	TP9525	CHE	NA	Breeding material
EUC_TP_109	TP9645	CHE	NA	Breeding material
EUC_TP_110	TP9445	CHE	NA	Breeding material
EUC_TP_111	TP9735	CHE	NA	Breeding material
EUC_TP_112	TP9315	CHE	NA	Breeding material
EUC_TP_113	Gumpensteiner Rotklee	AUT	1974	Cultivar
EUC_TP_114	Cinnamon Plus	USA	2003	Cultivar
EUC_TP_115	DFRC11	USA	NA	Breeding material
EUC_TP_116	DFRC12	USA	NA	Breeding material
EUC_TP_117	DFRC13	USA	NA	Breeding material
EUC_TP_118	DFRC14	USA	NA	Breeding material
EUC_TP_119	DFRC15	USA	NA	Breeding material
EUC_TP_120	FF 9615	USA	2013	Cultivar
EUC_TP_121	Marathon	USA	1985/1994	Cultivar
EUC_TP_122	Starfire I	USA	1999	Cultivar
EUC_TP_123	Starfire II	USA	2007	Cultivar
EUC_TP_124	GnRk0729	NOR	NA	Breeding material
EUC_TP_125	GnRk0747	NOR	NA	Breeding material
EUC_TP_126	KvRk0201	NOR	NA	Breeding material
EUC_TP_127	LGRk8801	NOR	NA	Breeding material
EUC_TP_128	LGRk9415	NOR	NA	Breeding material
EUC_TP_129	Linus	NOR	2014	Cultivar
EUC_TP_130	LøRk0286	NOR	NA	Breeding material
EUC_TP_131	LøRk0287	NOR	NA	Breeding material
EUC_TP_132	Linn	NOR	2021	Cultivar
EUC_TP_134	LøRk9207	NOR	NA	Breeding material
EUC_TP_135	LøRk9625	NOR	NA	Breeding material
EUC_TP_136	LøRk9627	NOR	NA	Breeding material

EUC_TP_137	LøRk9628	NOR	NA	Breeding material
EUC_TP_138	LøRk9753	NOR	NA	Breeding material
EUC_TP_139	VåRk0401	NOR	NA	Breeding material
EUC_TP_140	VåRk0510	NOR	NA	Breeding material
EUC_TP_141	VåRk0512	NOR	NA	Breeding material
EUC_TP_142	VåRk0513	NOR	NA	Breeding material
EUC_TP_143	VåRk0624	NOR	NA	Breeding material
EUC_TP_144	VåRk0625	NOR	NA	Breeding material
EUC_TP_145	K 39	SRB	NA	Cultivar
EUC_TP_146	Diplomat	DEU	2001	Cultivar
EUC_TP_147	SW 1479004	SWE	NA	Breeding material
EUC_TP_148	SW 1578301	SWE	NA	Breeding material
EUC_TP_149	SW 1678001	SWE	NA	Breeding material
EUC_TP_150	SW RK1092	SWE	NA	Breeding material
EUC_TP_151	SW RK1117	SWE	NA	Breeding material
EUC_TP_152	SW RK1118	SWE	NA	Breeding material
EUC_TP_153	SW RK1119	SWE	NA	Breeding material
EUC_TP_154	SW RK1120	SWE	NA	Breeding material
EUC_TP_155	SW RK1121	SWE	NA	Breeding material
EUC_TP_156	SW RK1122	SWE	NA	Breeding material
EUC_TP_157	SW RK1123	SWE	NA	Breeding material
EUC_TP_158	SW RK1124	SWE	NA	Breeding material
EUC_TP_159	SW RK1125	SWE	NA	Breeding material
EUC_TP_160	SW RK1131	SWE	NA	Breeding material
EUC_TP_161	SW RK1132	SWE	NA	Breeding material
EUC_TP_162	SW RK1133	SWE	NA	Breeding material
EUC_TP_163	SW RK1134	SWE	NA	Breeding material
EUC_TP_164	SW Yngve	SWE	2005	Cultivar
EUC_TP_165	SWÅ RK09093	SWE	NA	Breeding material
EUC_TP_166	0780MP2	CZE	2007	Breeding material
EUC_TP_167	08102MP2	CZE	2008	Breeding material
EUC_TP_168	08102MP4	CZE	2008	Breeding material
EUC_TP_169	Callisto	CZE	2011	Cultivar
EUC_TP_170	Elara	CZE	2014	Cultivar
EUC_TP_171	Ganymed	CZE	2016	Cultivar

EUC_TP_172	Hegemon	CZE	2017	Cultivar
EUC_TP_173	Helike	CZE	2016	Cultivar
EUC_TP_174	HŽ 2004 80 - 01	CZE	2004	Breeding material
EUC_TP_175	JL 2n 07 80 MP 1	CZE	2007	Breeding material
EUC_TP_176	Kalyke	CZE	2017	Cultivar
EUC_TP_177	TPD-05-11-18002	CZE	2011	Breeding material
EUC_TP_178	TPD-05-11-3087	CZE	2011	Breeding material
EUC_TP_179	TPD-05-11-3088	CZE	2011	Breeding material
EUC_TP_180	TPD-05-13-3080	CZE	2013	Breeding material
EUC_TP_181	TPD-05-13-3085	CZE	2013	Breeding material
EUC_TP_182	TPD-05-13-3091	CZE	2013	Breeding material
EUC_TP_183	TPD-05-14-1011	CZE	2014	Breeding material
EUC_TP_184	TPD-05-15-3127	CZE	2015	Breeding material
EUC_TP_185	TPD-05-15-3128	CZE	2015	Breeding material
EUC_TP_186	TPD-05-15-3129	CZE	2015	Breeding material
EUC_TP_187	TPD-05-16-3076	CZE	2016	Breeding material
EUC_TP_188	TPD-05-16-3146	CZE	2016	Breeding material
EUC_TP_189	TPD-05-16-3177	CZE	2016	Breeding material
EUC_TP_190	NA	FRA	1986	Cultivar
EUC_TP_191	NA	FRA	1984	Cultivar
EUC_TP_192	NA	FRA	2012	Cultivar
EUC_TP_193	NA	FRA	2010	Cultivar
EUC_TP_194	Grasslands Hamua	NZL	1946	Cultivar
EUC_TP_195	Grasslands Turoa	NZL	1932	Cultivar
EUC_TP_196	Relish	NZL	2012	Cultivar
EUC_TP_197	Ruby/Enterprise	NZL	~1990	Cultivar
EUC_TP_198	Natsuyu	JPN	2004	Cultivar
EUC_TP_199	Ryokuyu	JPN	2012	Cultivar
EUC_TP_200	NS-Mlava	SRB	2010	Cultivar
EUC_TP_201	NS-Petnica	SRB	2012	Cultivar
EUC_TP_202	NS-Sana	SRB	2014	Cultivar
EUC_TP_203	Una(NS)	SRB	2016	Cultivar
EUC_TP_204	Zoja (NS)	SRB	2012	Cultivar
EUC_TP_205	Avisto	BEL	2011	Cultivar
EUC_TP_206	Crossway	NZL	2002	Cultivar

EUC_TP_207	Lemmon	BEL	2000	Cultivar
EUC_TP_208	Merkemse	BEL	1955	Landrace
EUC_TP_209	Tp.12.12	BEL	NA	Breeding material
EUC_TP_210	Tp.14.7	BEL	NA	Breeding material
EUC_TP_211	Tandy	BEL	2021	Cultivar
EUC_TP_212	Agil	CZE	2009	Cultivar
EUC_TP_214	Brisk	CZE	2009	Cultivar
EUC_TP_215	Chlumecký	CZE	1935	Cultivar
EUC_TP_218	Feng	CZE	2015	Cultivar
EUC_TP_219	Garant	CZE	2008	Cultivar
EUC_TP_223	Respect	CZE	2010	Cultivar
EUC_TP_224	Slavín	CZE	2006	Cultivar
EUC_TP_225	Slavoj	CZE	2006	Cultivar
EUC_TP_227	Spurt	CZE	2009	Cultivar
EUC_TP_228	Start	CZE	1973	Cultivar
EUC_TP_229	Suez	CZE	2001	Cultivar
EUC_TP_231	Trubadur	CZE	2011	Cultivar
EUC_TP_232	Van	CZE	2012	Cultivar
EUC_TP_233	Vendelín	CZE	2005	Cultivar
EUC_TP_234	Vltavín	CZE	1992	Cultivar
EUC_TP_236	Zefyr	CZE	2014	Cultivar
EUC_TP_237	Harmonie	DEU	2007	Cultivar
EUC_TP_238	Regent	DEU	2008	Cultivar
EUC_TP_239	NGB1736	DNK	1964	Cultivar
EUC_TP_240	NGB2347	SWE	1970	Cultivar
EUC_TP_241	NGB2349	SWE	1941	Cultivar
EUC_TP_242	NGB2395	SWE	NA	Landrace
EUC_TP_243	NGB2452	SWE	NA	Landrace
EUC_TP_244	NGB2453	SWE	NA	Landrace
EUC_TP_245	NGB2464	SWE	NA	Landrace
EUC_TP_246	NGB2465	SWE	NA	Landrace
EUC_TP_247	NGB2466	SWE	NA	Landrace
EUC_TP_248	NGB2468	SWE	NA	Landrace
EUC_TP_249	NGB2469	SWE	NA	Landrace
EUC_TP_250	NGB2471	SWE	NA	Landrace

EUC_TP_251	NGB2472	SWE	NA	Landrace
EUC_TP_252	NGB2473	SWE	NA	Landrace
EUC_TP_253	NGB2474	SWE	NA	Landrace
EUC_TP_254	NGB2475	SWE	NA	Landrace
EUC_TP_255	NGB2476	SWE	NA	Landrace
EUC_TP_256	NGB2477	SWE	NA	Landrace
EUC_TP_257	NGB2481	SWE	NA	Landrace
EUC_TP_258	NGB2482	SWE	NA	Landrace
EUC_TP_259	NGB2494	SWE	NA	Landrace
EUC_TP_260	NGB2495	SWE	NA	Landrace
EUC_TP_261	NGB2569	SWE	NA	Landrace
EUC_TP_262	NGB2598	SWE	NA	Landrace
EUC_TP_263	NGB2599	SWE	NA	Landrace
EUC_TP_264	NGB2600	SWE	NA	Landrace
EUC_TP_265	NGB2739	SWE	1937	Cultivar
EUC_TP_266	NGB2740	SWE	1965	Cultivar
EUC_TP_267	NGB2742	SWE	1957	Cultivar
EUC_TP_268	NGB2745	SWE	1977	Cultivar
EUC_TP_269	NGB2746	SWE	1974	Cultivar
EUC_TP_270	NGB2747	SWE	1970	Cultivar
EUC_TP_271	NGB2748	SWE	1951	Cultivar
EUC_TP_272	NGB2749	SWE	1970	Cultivar
EUC_TP_273	NGB2750	SWE	1960	Cultivar
EUC_TP_274	NGB2751	SWE	1976	Cultivar
EUC_TP_275	NGB4126	DNK	1970	Cultivar
EUC_TP_277	NGB7510	SWE	NA	Cultivar
EUC_TP_278	NGB9966	SWE	NA	Landrace
EUC_TP_279	Diadem	FRA	2002	Cultivar
EUC_TP_280	Diper	FRA	1980	Cultivar
EUC_TP_281	Diplo	FRA	2006	Cultivar
EUC_TP_282	Kindia	FRA	2006	Cultivar
EUC_TP_283	Affoltern i.E._325	CHE	1972	Landrace
EUC_TP_284	Bern_76	CHE	1972	Landrace
EUC_TP_285	Bigenthal_163	CHE	1972	Landrace
EUC_TP_286	Biglen_352	CHE	1972	Landrace

EUC_TP_287	Englisberg_249	CHE	1972	Landrace		
EUC_TP_288	Grossdietwil_21	CHE	1972	Landrace		
EUC_TP_289	Haeusernmoos_333	CHE	1972	Landrace		
EUC_TP_290	Koeniz_239	CHE	1972	Landrace		
EUC_TP_291	Koeniz_247	CHE	1972	Landrace		
EUC_TP_292	Lauperswil_138	CHE	1972	Landrace		
EUC_TP_293	MontCalme	CHE	1970	Cultivar		
EUC_TP_294	Neuenegg_340	CHE	1972	Landrace		
EUC_TP_295	Niederscherli_273	CHE	1972	Landrace		
EUC_TP_296	Oberbottigen_7	CHE	1972	Landrace		
EUC_TP_297	Oberoenz_321	CHE	1972	Landrace		
EUC_TP_298	Oeschenbach_330	CHE	1972	Landrace		
EUC_TP_299	Renova	CHE	1964	Cultivar		
EUC_TP_300	Riggisberg_318	CHE	1972	Landrace		
EUC_TP_301	Rueedisbach_332	CHE	1972	Landrace		
EUC_TP_302	Rüttinova	CHE	1984	Cultivar		
EUC_TP_303	Schmitten_5	CHE	1972	Landrace		
EUC_TP_304	Signau_154	CHE	1972	Landrace		
EUC_TP_305	Wasen i.E._199	CHE	1972	Landrace		
EUC_TP_306	Weier i.E._327	CHE	1972	Landrace		
EUC_TP_307	Aa 3090	GBR	1948	Cultivar		
EUC_TP_308	Aa 3108	GBR	1948	Cultivar		
EUC_TP_310	Aa 4189	POL	1990	Ecotype	49.25	19.95
EUC_TP_311	Aa 4297	CZE	1992	Ecotype	49.483	18.2667
EUC_TP_312	Aa 4398	GBR	1996	Ecotype	54.683	-2.2667
EUC_TP_313	Aa 4403	GBR	1996	Ecotype	51.233	-2.6833
EUC_TP_315	Aa 4445	ITA	1998	Ecotype	46.05	12.8
EUC_TP_316	Aa 4448	ITA	1998	Ecotype	45.767	13
EUC_TP_317	Aa 4456	ITA	1998	Ecotype	45.85	12.8
EUC_TP_318	Aa 4515	ESP	2003	Ecotype	43.328	-4.8786
EUC_TP_319	Aa 4520	ESP	2005	Ecotype	43.128	-5.8141
EUC_TP_320	Aa 4525	ESP	2003	Ecotype	43.379	-5.8658
EUC_TP_321	Aa 4527	ESP	2003	Ecotype	40.517	-5.2695
EUC_TP_322	Aa 4529	ESP	2003	Ecotype	43.276	-5.8084
EUC_TP_324	Aa 4593	GBR	2009	Cultivar		

EUC_TP_325	Aa 4934	GBR	2012	Breeding material
EUC_TP_326	Aa 4936	GBR	2012	Breeding material
EUC_TP_327	Aa 4937	GBR	2012	Breeding material
EUC_TP_328	Aa 5417	GBR	2015	Breeding material
EUC_TP_329	SW 1479001	SWE	NA	Breeding material
EUC_TP_330	SW 1479002	SWE	NA	Breeding material
EUC_TP_331	SW 1479003	SWE	NA	Breeding material
EUC_TP_332	SW 1678002	SWE	NA	Breeding material
EUC_TP_333	SW 1678003	SWE	NA	Breeding material
EUC_TP_334	SW 1678004	SWE	NA	Breeding material
EUC_TP_335	SW 1678401	SWE	NA	Breeding material
EUC_TP_336	SW 1678402	SWE	NA	Breeding material
EUC_TP_337	SW 1678403	SWE	NA	Breeding material
EUC_TP_338	SW RK1095	SWE	NA	Breeding material
EUC_TP_339	SW RK1096	SWE	NA	Breeding material
EUC_TP_340	SW RK1097	SWE	NA	Breeding material
EUC_TP_341	SW RK1102	SWE	NA	Breeding material
EUC_TP_342	SW RK1160	SWE	NA	Breeding material
EUC_TP_343	SW RK1161	SWE	NA	Breeding material
EUC_TP_344	SW RK1162	SWE	NA	Breeding material
EUC_TP_345	SW RK1164	SWE	NA	Breeding material
EUC_TP_346	SWA 1376104	SWE	NA	Breeding material
EUC_TP_347	SWA 1376105	SWE	NA	Breeding material
EUC_TP_348	SWA 1476014	SWE	NA	Breeding material
EUC_TP_349	SWA 1476016	SWE	NA	Breeding material
EUC_TP_350	SWA 1476017	SWE	NA	Breeding material
EUC_TP_351	SWA 1476019	SWE	NA	Breeding material
EUC_TP_352	SWA 1575301	SWE	NA	Breeding material
EUC_TP_353	SWA 1575302	SWE	NA	Breeding material
EUC_TP_354	SWA 1575304	SWE	NA	Breeding material
EUC_TP_355	SWA 1575305	SWE	NA	Breeding material
EUC_TP_356	SWA 1575306	SWE	NA	Breeding material
EUC_TP_357	SWA 1575307	SWE	NA	Breeding material
EUC_TP_358	SWA 1575308	SWE	NA	Breeding material
EUC_TP_359	SWA 1575309	SWE	NA	Breeding material

EUC_TP_360	SWA 1575312	SWE	NA	Breeding material		
EUC_TP_361	SWA 1576005	SWE	NA	Breeding material		
EUC_TP_362	SWA 1675205	SWE	NA	Breeding material		
EUC_TP_363	SWA 1675206	SWE	NA	Breeding material		
EUC_TP_364	SWA 1675207	SWE	NA	Breeding material		
EUC_TP_365	SWA 1675208	SWE	NA	Breeding material		
EUC_TP_366	SWA 1675212	SWE	NA	Breeding material		
EUC_TP_367	TPD-05-04-3000	CZE	2004	Breeding material		
EUC_TP_368	TPD-05-11-3007	CZE	2011	Breeding material		
EUC_TP_369	TPD-05-12-3018	CZE	2012	Breeding material		
EUC_TP_370	Avala (NS)	SRB	2008	Cultivar		
EUC_TP_371	BL-1-Banja Luka	SRB	1996	Breeding material		
EUC_TP_372	BL-3-Banja Luka	SRB	1996	Breeding material		
EUC_TP_373	BL-4-Banja Luka	SRB	1996	Breeding material		
EUC_TP_374	BL-5-Banja Luka	SRB	1996	Breeding material		
EUC_TP_375	D-1	SRB	2008	Breeding material		
EUC_TP_376	D-10	SRB	2008	Breeding material		
EUC_TP_377	D-2	SRB	2008	Breeding material		
EUC_TP_378	D-3	SRB	2008	Breeding material		
EUC_TP_379	D-4	SRB	2008	Breeding material		
EUC_TP_380	D-5	SRB	2008	Breeding material		
EUC_TP_381	D-6	SRB	2008	Breeding material		
EUC_TP_382	D-7	SRB	2008	Breeding material		
EUC_TP_383	D-8	SRB	2008	Breeding material		
EUC_TP_384	D-9	SRB	2008	Breeding material		
EUC_TP_385	M10-Kopaonik	SRB	1996	Ecotype	NA	NA
EUC_TP_386	M11-Kopaonik	SRB	1996	Ecotype	NA	NA
EUC_TP_387	M12-Kopaonik	SRB	1996	Ecotype	NA	NA
EUC_TP_388	M13-Kopaonik	SRB	1996	Ecotype	NA	NA
EUC_TP_389	M14-Kopaonik	SRB	1996	Ecotype	NA	NA
EUC_TP_390	NS-Ravanica	SRB	2010	Cultivar		
EUC_TP_391	Broadway	NZL	2002	Cultivar		
EUC_TP_392	Kontiki	DEU	2010	Cultivar		
EUC_TP_393	Mercury	BEL	1994	Cultivar		
EUC_TP_394	Merian	BEL	2000	Cultivar		

EUC_TP_395	Oudenaerdse	BEL	1950	Landrace
EUC_TP_396	Primus	BEL	1967	Landrace
EUC_TP_397	Tp.08.4	BEL	NA	Breeding material
EUC_TP_398	Tp.08.5	BEL	NA	Breeding material
EUC_TP_399	Violetta	BEL	1954	Cultivar
EUC_TP_400	Waesse	BEL	1950	Landrace
EUC_TP_446	Affoltern i.E._186	CHE	1972	Landrace
EUC_TP_447	Arni b.Biglen_351	CHE	1972	Landrace
EUC_TP_449	Horgen_1	CHE	1972	Landrace
EUC_TP_454	Lanzenhaeusern_292	CHE	1972	Landrace
EUC_TP_456	Riggisberg_311	CHE	1972	Landrace
EUC_TP_495[a]	TP1035	CHE	2010	Breeding material
EUC_TP_496[a]	TP1115	CHE	2011	Breeding material
EUC_TP_497[a]	TP1135	CHE	2011	Breeding material
EUC_TP_498[a]	TP1205	CHE	2012	Breeding material
EUC_TP_499[a]	TP1305	CHE	2013	Breeding material
EUC_TP_660	LøRk0498	NOR	NA	Breeding material
EUC_TP_661	SWA 1575303	SWE	NA	Breeding material
EUC_TP_662	SWA 1576001	SWE	NA	Breeding material

[a] not included in the clover rot resistance trial
