# Supplementary material for: Phenotypic variation and quantitative trait loci for resistance to southern anthracnose and clover rot in red clover
Source: Theor Appl Genet. 2022 Sep 25;135(12):4337–49. doi: 10.1007/s00122-022-04223-8 (PMC9734235; doi:10.1007/s00122-022-04223-8)
Supplement: Supplementary file 4 — Supplementary file4 (PDF 343 kb) [file 122_2022_4223_MOESM4_ESM.pdf]

Supplementary Table 2 Accession ID, mean survival rate (%) for southern anthracnose single-spore inoculation, cumulative survival rate (%) for southern anthracnose mixed-spore inoculation and mean resistance index (%) for cover rot as well as standard error (SE) for the three traits and all accessions involved in this study

EUCLEG ID	Southern anthracnose mean survivor rate (%) single-spore inoculation	SE single-spore inoculation	Southern anthracnose mean cumulative survival rate (%) mixed-spore inoculation[ab]	SE mixed-spore inoculation[a]	Clover rot mean resistance index (%)	SE of resistance index
EUC_TP_001	16.84	5.61	10.92	0.67	33.72	3.37
EUC_TP_002	9.87	5.62	2.63	0.67	37.31	3.34
EUC_TP_003	16.29	5.61	8.48	0.67	35.71	3.35
EUC_TP_004	20.61	0.96	8.72	0.11	36.72	3.24
EUC_TP_005	43.66	0.96	29.93	0.11	29.07	3.35
EUC_TP_006	17.40	5.62	9.18	0.67	36.26	3.35
EUC_TP_007	22.25	5.62	13.80	0.67	33.91	3.35
EUC_TP_008	20.64	5.62	6.14	0.67	39.48	3.38
EUC_TP_009	13.82	5.62	7.08	0.67	41.46	3.35
EUC_TP_010	18.98	6.48	1.31	0.78	25.02	3.35
EUC_TP_011	14.55	5.62	4.21	0.67	27.07	3.40
EUC_TP_012	23.55	5.62	12.60	0.67	40.32	3.35
EUC_TP_013	21.82	5.62	14.52	0.67	39.77	3.34
EUC_TP_014	57.19	5.62	49.77	0.67	35.19	3.36
EUC_TP_015	20.03	5.62	13.53	0.67	37.75	3.35
EUC_TP_016	29.90	5.62	17.00	0.67	29.32	3.34
EUC_TP_017	12.95	5.62	3.88	0.67	36.14	3.35
EUC_TP_018	39.17	5.62	22.59	0.67	34.35	3.16
EUC_TP_019	17.80	5.62	4.70	0.67	37.45	3.36
EUC_TP_020	42.89	5.61	30.18	0.67	33.24	3.34
EUC_TP_021	12.20	6.48	3.81	0.78	39.84	3.39
EUC_TP_022	4.95	5.62	1.77	0.67	35.66	3.25
EUC_TP_023	11.32	5.62	6.25	0.67	39.17	3.34
EUC_TP_024	30.69	6.48	16.64	0.78	45.91	3.27
EUC_TP_025	19.54	5.62	9.15	0.67	34.21	3.34
EUC_TP_026	14.09	5.62	7.02	0.67	35.66	3.34
EUC_TP_027	23.18	5.62	9.03	0.67	32.99	3.37
EUC_TP_028	13.24	5.61	4.18	0.67	42.33	3.41
EUC_TP_029	20.50	5.62	5.81	0.67	28.68	3.43
EUC_TP_030	13.05	5.62	3.96	0.67	34.37	3.35
EUC_TP_031	42.88	5.62	36.66	0.67	43.36	3.35
EUC_TP_032	20.22	5.62	5.01	0.67	39.84	3.34
EUC_TP_033	18.30	5.62	10.92	0.67	35.89	3.35
EUC_TP_034	11.88	5.62	4.74	0.67	30.90	3.36
EUC_TP_035	16.06	5.61	5.18	0.67	30.18	3.24
EUC_TP_036	10.63	5.61	0.52	0.67	36.41	3.34
EUC_TP_037	18.45	5.61	4.04	0.67	33.47	3.23

EUC_TP_038	14.26	5.62	2.79	0.67	29.88	3.35
EUC_TP_039	7.72	5.61	1.83	0.67	34.77	3.35
EUC_TP_040	9.54	5.62	4.70	0.67	27.38	3.34
EUC_TP_041	8.96	5.62	1.35	0.67	32.89	3.34
EUC_TP_042	11.95	5.62	1.49	0.67	31.02	3.34
EUC_TP_043	7.27	5.62	1.16	0.67	28.87	3.37
EUC_TP_044	19.73	5.61	10.63	0.67	29.86	3.35
EUC_TP_045	34.08	5.62	20.63	0.67	30.36	3.35
EUC_TP_046	17.61	5.61	7.37	0.67	30.03	3.24
EUC_TP_047	52.63	5.61	34.23	0.67	34.11	3.38
EUC_TP_048	20.64	5.62	10.12	0.67	32.52	3.42
EUC_TP_049	49.89	5.61	44.29	0.67	38.58	3.34
EUC_TP_050	4.65	5.62	0.43	0.67	26.76	3.36
EUC_TP_051	14.47	5.62	4.30	0.67	30.59	3.34
EUC_TP_052	8.88	5.62	0.40	0.67	27.07	3.40
EUC_TP_053	5.42	5.62	0.60	0.67	28.53	3.39
EUC_TP_054	11.35	5.61	1.46	0.67	30.99	3.35
EUC_TP_055	9.06	5.61	1.37	0.67	29.38	3.36
EUC_TP_056	4.47	5.62	2.05	0.67	28.06	3.34
EUC_TP_057	18.59	5.62	9.62	0.67	27.31	3.35
EUC_TP_058	52.02	5.61	41.64	0.67	30.89	3.26
EUC_TP_059	36.32	5.61	27.36	0.67	35.00	3.36
EUC_TP_060	53.84	5.62	35.75	0.67	31.12	3.35
EUC_TP_061	9.96	5.62	4.86	0.67	37.15	3.34
EUC_TP_062	10.39	5.62	4.63	0.67	28.62	3.34
EUC_TP_063	8.43	5.61	5.15	0.67	36.94	3.37
EUC_TP_064	13.04	5.62	3.11	0.67	28.89	3.34
EUC_TP_065	12.34	5.62	4.89	0.67	29.87	3.35
EUC_TP_066	25.34	5.62	12.38	0.67	34.35	3.23
EUC_TP_067	18.63	5.62	9.00	0.67	30.86	3.33
EUC_TP_068	11.32	5.62	3.32	0.67	25.58	3.36
EUC_TP_069	17.60	5.62	0.72	0.67	32.59	3.35
EUC_TP_070	14.08	5.62	10.45	0.67	29.34	3.34
EUC_TP_071	57.19	5.61	39.51	0.67	41.87	3.35
EUC_TP_072	9.69	5.61	3.29	0.67	33.55	3.35
EUC_TP_073	6.30	5.61	1.70	0.67	26.60	3.34
EUC_TP_074	9.71	5.62	1.71	0.67	37.14	3.37
EUC_TP_075	15.19	5.61	2.59	0.67	33.24	3.34
EUC_TP_076	8.09	5.62	4.04	0.67	24.86	3.34
EUC_TP_077	5.60	5.62	4.35	0.67	33.06	3.36
EUC_TP_078	13.02	5.62	2.99	0.67	26.53	3.34
EUC_TP_079	8.59	5.62	1.72	0.67	24.96	3.37
EUC_TP_080	8.49	5.62	1.93	0.67	26.84	3.35
EUC_TP_081	10.94	5.62	0.30	0.67	30.70	3.39
EUC_TP_082	5.22	5.62	1.19	0.67	22.18	3.34
EUC_TP_083	5.21	5.62	0.00	0.67	28.43	3.34
EUC_TP_084	12.51	5.62	0.54	0.67	41.95	3.25

EUC_TP_085	13.97	5.62	5.57	0.67	25.25	3.36
EUC_TP_086	10.30	5.62	0.18	0.67	30.08	3.34
EUC_TP_087	46.33	5.62	35.93	0.67	30.34	3.35
EUC_TP_088	17.87	5.61	4.42	0.67	33.86	3.36
EUC_TP_089	3.89	5.62	0.00	0.67	30.97	3.35
EUC_TP_090	26.62	5.62	20.65	0.67	38.56	3.35
EUC_TP_091	2.56	5.61	0.66	0.67	23.46	3.35
EUC_TP_092	5.86	5.61	0.01	0.67	33.77	3.34
EUC_TP_093	21.46	5.62	18.55	0.67	41.89	3.23
EUC_TP_094	19.92	5.62	5.70	0.67	25.36	3.40
EUC_TP_095	16.36	5.61	11.01	0.67	26.76	3.35
EUC_TP_097	6.78	5.62	4.88	0.67	26.74	3.35
EUC_TP_098	26.51	5.62	14.30	0.67	27.88	3.35
EUC_TP_099	58.06	5.62	42.47	0.67	31.93	3.35
EUC_TP_100	57.19	5.62	49.70	0.67	32.66	3.34
EUC_TP_101	12.74	5.62	4.85	0.67	23.03	3.26
EUC_TP_102	42.38	5.62	34.03	0.67	32.63	3.24
EUC_TP_103	40.62	5.62	32.50	0.67	31.97	3.35
EUC_TP_104	18.21	5.62	8.54	0.67	41.15	3.34
EUC_TP_105	12.82	5.61	4.78	0.67	32.67	3.34
EUC_TP_106	12.63	5.62	0.62	0.67	28.71	3.25
EUC_TP_108	17.00	5.62	3.75	0.67	37.62	3.23
EUC_TP_109	31.27	5.62	18.88	0.67	37.97	3.34
EUC_TP_110	45.66	5.61	30.98	0.67	36.54	3.36
EUC_TP_111	52.97	5.62	43.74	0.67	37.21	3.24
EUC_TP_112	38.11	5.61	23.60	0.67	42.06	3.27
EUC_TP_113	8.88	5.62	0.33	0.67	28.18	3.40
EUC_TP_114	73.93	5.62	65.09	0.67	43.69	3.35
EUC_TP_115	51.57	5.62	44.25	0.67	21.22	3.34
EUC_TP_116	70.41	5.62	57.51	0.67	30.23	3.23
EUC_TP_117	56.11	5.62	48.87	0.67	31.13	3.34
EUC_TP_118	57.42	5.62	49.35	0.67	32.47	3.37
EUC_TP_119	67.25	5.62	45.04	0.67	32.30	3.34
EUC_TP_120	53.38	5.62	42.28	0.67	35.83	3.23
EUC_TP_121	50.79	6.48	43.04	0.78	26.35	3.34
EUC_TP_122	56.01	5.62	49.82	0.67	30.29	3.34
EUC_TP_123	62.57	5.62	55.96	0.67	29.19	3.34
EUC_TP_124	29.73	5.62	18.62	0.67	40.25	3.33
EUC_TP_125	11.25	5.62	6.21	0.67	44.17	3.34
EUC_TP_126	20.78	5.62	12.12	0.67	37.08	3.36
EUC_TP_127	33.21	5.62	28.56	0.67	37.23	3.37
EUC_TP_128	27.01	5.61	15.00	0.67	41.49	3.35
EUC_TP_129	16.94	5.62	9.83	0.67	29.62	3.23
EUC_TP_130	39.52	5.62	27.47	0.67	37.54	3.35
EUC_TP_131	16.27	5.62	4.62	0.67	33.31	3.35
EUC_TP_132	29.73	5.62	18.79	0.67	34.30	3.35
EUC_TP_134	19.84	5.62	12.30	0.67	39.70	3.34

EUC_TP_135	37.24	5.62	30.34	0.67	41.67	3.37
EUC_TP_136	16.55	5.61	6.56	0.67	30.51	3.34
EUC_TP_137	20.66	5.62	8.76	0.67	43.04	3.37
EUC_TP_138	29.70	5.62	17.51	0.67	38.51	3.34
EUC_TP_139	45.83	5.61	25.32	0.67	40.11	3.34
EUC_TP_140	24.29	5.62	9.98	0.67	28.17	3.35
EUC_TP_141	32.90	5.62	21.33	0.67	31.74	3.23
EUC_TP_142	31.77	5.61	19.14	0.67	38.08	3.35
EUC_TP_143	25.32	5.62	18.74	0.67	44.63	3.34
EUC_TP_144	40.63	5.62	27.41	0.67	39.07	3.35
EUC_TP_145	17.32	5.62	4.73	0.67	27.39	3.37
EUC_TP_146	18.68	5.62	0.96	0.67	30.77	3.34
EUC_TP_147	22.16	5.62	5.70	0.67	31.06	3.35
EUC_TP_148	11.13	5.61	5.24	0.67	32.41	3.23
EUC_TP_149	17.51	5.62	10.40	0.67	34.93	3.36
EUC_TP_150	20.24	5.62	6.87	0.67	38.66	3.34
EUC_TP_151	23.25	5.62	16.08	0.67	36.56	3.34
EUC_TP_152	25.31	5.62	13.34	0.67	38.01	3.34
EUC_TP_153	15.17	5.62	6.55	0.67	30.58	3.35
EUC_TP_154	23.49	5.62	10.18	0.67	34.81	3.35
EUC_TP_155	13.20	5.62	6.75	0.67	37.44	3.39
EUC_TP_156	26.36	5.62	13.50	0.67	38.79	3.24
EUC_TP_157	21.01	5.61	11.19	0.67	35.62	3.35
EUC_TP_158	18.14	5.61	7.64	0.67	32.56	3.35
EUC_TP_159	22.17	5.61	15.13	0.67	39.17	3.24
EUC_TP_160	24.15	5.62	14.93	0.67	39.22	3.35
EUC_TP_161	14.10	5.62	2.58	0.67	38.03	3.35
EUC_TP_162	28.74	5.62	20.74	0.67	35.86	3.23
EUC_TP_163	24.86	5.62	17.06	0.67	38.31	3.35
EUC_TP_164	10.42	5.61	6.91	0.67	36.30	3.36
EUC_TP_165	15.19	5.61	5.56	0.67	43.17	3.34
EUC_TP_166	35.05	5.62	18.94	0.67	30.78	3.36
EUC_TP_167	24.19	5.62	18.38	0.67	37.92	3.36
EUC_TP_168	27.25	5.62	12.62	0.67	41.85	3.34
EUC_TP_169	30.15	5.62	14.93	0.67	38.77	3.35
EUC_TP_170	23.73	5.62	15.95	0.67	31.29	3.35
EUC_TP_171	25.83	5.62	19.59	0.67	34.05	3.34
EUC_TP_172	42.77	5.62	27.62	0.67	29.40	3.35
EUC_TP_173	19.84	5.61	8.76	0.67	30.62	3.35
EUC_TP_174	14.23	5.61	4.26	0.67	42.51	3.38
EUC_TP_175	12.71	5.62	1.43	0.67	42.98	3.35
EUC_TP_176	22.36	5.62	17.55	0.67	40.35	3.24
EUC_TP_177	30.46	6.48	9.81	0.78	32.40	3.39
EUC_TP_178	19.68	5.61	3.39	0.67	41.07	3.34
EUC_TP_179	21.67	5.61	11.48	0.67	40.06	3.24
EUC_TP_180	38.38	5.62	29.13	0.67	34.09	3.34
EUC_TP_181	27.06	5.62	13.12	0.67	40.81	3.23

EUC_TP_182	55.60	5.62	31.48	0.67	42.49	3.36
EUC_TP_183	17.95	5.62	6.33	0.67	30.11	3.34
EUC_TP_184	50.25	5.62	45.88	0.67	44.00	3.33
EUC_TP_185	42.45	5.62	22.91	0.67	32.99	3.34
EUC_TP_186	38.23	5.62	25.33	0.67	43.06	3.36
EUC_TP_187	46.03	5.62	31.99	0.67	34.61	3.34
EUC_TP_188	38.81	5.61	31.68	0.67	38.32	3.36
EUC_TP_189	31.81	5.62	17.57	0.67	29.19	3.35
EUC_TP_190	20.05	5.62	9.50	0.67	30.47	3.34
EUC_TP_191	21.47	5.62	9.67	0.67	39.32	3.34
EUC_TP_192	17.73	5.61	6.79	0.67	37.61	3.24
EUC_TP_193	16.42	5.62	9.42	0.67	36.87	3.35
EUC_TP_194	11.80	5.62	4.43	0.67	27.29	3.38
EUC_TP_195	9.92	5.61	0.04	0.67	35.98	3.53
EUC_TP_196	22.54	5.62	16.55	0.67	34.86	3.35
EUC_TP_197	27.16	5.62	11.86	0.67	29.61	3.45
EUC_TP_198	41.40	5.62	32.10	0.67	38.26	3.36
EUC_TP_199	29.75	5.62	20.55	0.67	37.15	3.23
EUC_TP_200	38.54	5.62	27.78	0.67	29.81	3.35
EUC_TP_201	40.74	5.62	27.51	0.67	30.27	3.24
EUC_TP_202	40.42	5.62	32.49	0.67	31.67	3.23
EUC_TP_203	39.48	5.62	19.08	0.67	27.81	3.23
EUC_TP_204	38.00	5.62	19.85	0.67	31.60	3.35
EUC_TP_205	38.23	5.62	28.83	0.67	28.02	3.37
EUC_TP_206	23.45	5.61	18.33	0.67	23.09	3.36
EUC_TP_207	27.08	5.61	17.37	0.67	36.76	3.35
EUC_TP_208	10.78	5.62	1.09	0.67	31.02	3.23
EUC_TP_209	37.07	5.62	23.40	0.67	24.06	3.37
EUC_TP_210	14.91	5.62	7.85	0.67	34.21	3.34
EUC_TP_211	30.99	5.62	19.23	0.67	42.70	3.35
EUC_TP_212	72.70	5.62	63.72	0.67	36.64	3.34
EUC_TP_214	16.36	5.62	1.75	0.67	33.40	3.34
EUC_TP_215	14.19	6.48	2.34	0.78	33.97	3.35
EUC_TP_218	51.25	5.61	34.45	0.67	35.13	3.36
EUC_TP_219	39.90	5.62	22.44	0.67	35.49	3.34
EUC_TP_223	38.98	5.62	27.43	0.67	32.10	3.34
EUC_TP_224	17.46	6.48	5.22	0.78	36.54	3.35
EUC_TP_225	15.62	5.62	5.82	0.67	27.80	3.35
EUC_TP_227	39.00	5.62	25.08	0.67	29.98	3.34
EUC_TP_228	28.11	5.61	15.98	0.67	30.47	3.37
EUC_TP_229	12.55	5.62	6.49	0.67	34.43	3.35
EUC_TP_231	37.58	6.48	28.62	0.78	38.73	3.35
EUC_TP_232	55.11	5.62	53.67	0.67	36.18	3.48
EUC_TP_233	8.86	5.62	3.50	0.67	35.00	3.23
EUC_TP_234	10.79	5.61	2.28	0.67	30.61	3.34
EUC_TP_236	27.62	5.61	16.90	0.67	38.20	3.34
EUC_TP_237	30.63	5.62	11.66	0.67	37.02	3.34

EUC_TP_238	19.06	5.62	7.92	0.67	36.46	3.35
EUC_TP_239	20.53	5.62	13.22	0.67	29.03	3.37
EUC_TP_240	34.69	5.62	21.29	0.67	38.52	3.35
EUC_TP_241	26.80	5.62	17.96	0.67	29.46	3.35
EUC_TP_242	19.27	5.62	12.56	0.67	30.95	3.35
EUC_TP_243	15.60	5.62	2.71	0.67	31.64	3.35
EUC_TP_244	9.84	5.62	2.58	0.67	30.52	3.34
EUC_TP_245	11.87	5.62	5.76	0.67	35.69	3.34
EUC_TP_246	9.45	5.61	6.01	0.67	26.99	3.36
EUC_TP_247	20.45	5.61	3.60	0.67	28.12	3.23
EUC_TP_248	14.46	5.62	1.38	0.67	40.70	3.34
EUC_TP_249	7.76	5.62	2.59	0.67	32.96	3.35
EUC_TP_250	15.74	5.61	7.88	0.67	42.26	3.34
EUC_TP_251	11.44	5.62	5.23	0.67	39.40	3.34
EUC_TP_252	17.01	5.61	3.93	0.67	35.65	3.34
EUC_TP_253	12.77	5.62	1.80	0.67	32.91	3.24
EUC_TP_254	16.62	5.62	5.65	0.67	39.60	3.34
EUC_TP_255	13.65	5.62	6.13	0.67	38.42	3.35
EUC_TP_256	14.55	5.62	5.97	0.67	34.63	3.23
EUC_TP_257	13.41	5.62	1.99	0.67	33.54	3.34
EUC_TP_258	10.03	5.62	2.59	0.67	33.23	3.34
EUC_TP_259	7.94	5.62	0.45	0.67	38.18	3.35
EUC_TP_260	15.81	5.62	2.28	0.67	32.38	3.23
EUC_TP_261	15.81	5.62	5.25	0.67	34.45	3.35
EUC_TP_262	15.95	5.62	5.54	0.67	29.37	3.23
EUC_TP_263	12.84	5.62	8.10	0.67	29.22	3.23
EUC_TP_264	14.55	5.62	5.75	0.67	31.01	3.33
EUC_TP_265	24.66	5.62	16.40	0.67	38.77	3.34
EUC_TP_266	10.71	5.62	3.11	0.67	36.86	3.23
EUC_TP_267	15.74	6.48	6.84	0.78	39.86	3.24
EUC_TP_268	25.85	5.62	11.30	0.67	38.12	3.34
EUC_TP_269	20.64	5.61	7.88	0.67	37.98	3.25
EUC_TP_270	13.08	5.62	3.70	0.67	36.76	3.28
EUC_TP_271	10.54	5.62	5.78	0.67	32.42	3.33
EUC_TP_272	15.45	5.62	6.36	0.67	32.64	3.34
EUC_TP_273	5.57	5.62	3.79	0.67	28.96	3.35
EUC_TP_274	10.45	5.62	1.04	0.67	38.44	3.25
EUC_TP_275	23.35	5.62	13.08	0.67	34.42	3.38
EUC_TP_277	30.36	5.61	21.00	0.67	33.16	3.36
EUC_TP_278	17.32	6.48	6.22	0.78	37.89	3.35
EUC_TP_279	20.45	6.48	10.60	0.78	35.96	3.36
EUC_TP_280	20.38	5.61	9.11	0.67	39.39	3.35
EUC_TP_281	16.85	5.62	3.37	0.67	33.61	3.39
EUC_TP_282	5.02	5.62	0.97	0.67	43.34	3.35
EUC_TP_283	25.12	5.62	1.74	0.67	37.88	3.38
EUC_TP_284	18.95	5.61	7.30	0.67	33.68	3.25
EUC_TP_285	10.99	5.62	2.20	0.67	27.95	3.37

EUC_TP_286	19.51	5.62	1.56	0.67	35.23	3.39
EUC_TP_287	12.20	5.61	8.11	0.67	29.43	3.36
EUC_TP_288	13.19	5.62	10.62	0.67	33.94	3.38
EUC_TP_289	15.76	5.62	11.38	0.67	27.29	3.39
EUC_TP_290	10.86	5.62	2.85	0.67	32.02	3.35
EUC_TP_291	13.76	5.62	6.34	0.67	25.32	3.36
EUC_TP_292	17.58	5.62	3.92	0.67	31.88	3.34
EUC_TP_293	13.97	5.61	1.63	0.67	32.99	3.34
EUC_TP_294	9.89	5.61	1.52	0.67	32.89	3.36
EUC_TP_295	7.34	5.62	0.30	0.67	29.07	3.24
EUC_TP_296	8.25	5.61	0.98	0.67	35.60	3.35
EUC_TP_297	6.20	5.62	2.53	0.67	25.04	3.39
EUC_TP_298	7.00	5.61	0.99	0.67	34.55	3.25
EUC_TP_299	45.08	5.61	25.16	0.67	31.73	3.23
EUC_TP_300	16.67	5.62	1.50	0.67	38.92	3.43
EUC_TP_301	7.87	5.62	2.19	0.67	33.65	3.42
EUC_TP_302	22.02	5.62	9.30	0.67	36.24	3.37
EUC_TP_303	10.01	5.62	1.27	0.67	28.46	3.36
EUC_TP_304	15.00	5.61	3.61	0.67	25.04	3.35
EUC_TP_305	12.97	5.62	4.81	0.67	31.32	3.42
EUC_TP_306	12.34	6.48	2.40	0.78	31.43	3.30
EUC_TP_307	-0.37	5.62	0.02	0.67	27.76	3.34
EUC_TP_308	7.53	5.62	1.33	0.67	30.15	3.34
EUC_TP_310	10.84	5.61	5.93	0.67	37.71	3.36
EUC_TP_311	6.04	5.61	0.46	0.67	26.74	3.35
EUC_TP_312	9.00	5.62	2.33	0.67	33.82	3.24
EUC_TP_313	23.39	5.62	13.68	0.67	26.27	3.33
EUC_TP_315	14.36	5.62	9.12	0.67	35.03	3.26
EUC_TP_316	25.25	5.62	7.85	0.67	30.80	3.34
EUC_TP_317	39.44	5.62	31.39	0.67	35.38	3.24
EUC_TP_318	29.86	5.61	19.11	0.67	20.85	3.35
EUC_TP_319	1.47	5.62	1.53	0.67	28.39	3.24
EUC_TP_320	26.39	5.61	15.76	0.67	23.91	3.34
EUC_TP_321	28.30	5.62	17.52	0.67	24.46	3.35
EUC_TP_322	24.42	5.61	13.10	0.67	25.50	3.35
EUC_TP_324	12.89	5.61	9.19	0.67	33.18	3.24
EUC_TP_325	25.11	5.62	10.30	0.67	27.44	3.36
EUC_TP_326	30.95	5.62	8.67	0.67	38.00	3.35
EUC_TP_327	26.76	5.62	21.83	0.67	31.01	3.34
EUC_TP_328	6.29	5.62	2.29	0.67	32.28	3.23
EUC_TP_329	18.61	5.62	13.88	0.67	40.36	3.34
EUC_TP_330	25.94	5.61	14.96	0.67	44.42	3.24
EUC_TP_331	11.06	5.61	1.45	0.67	37.18	3.34
EUC_TP_332	27.49	5.62	17.46	0.67	29.99	3.35
EUC_TP_333	31.85	5.62	19.50	0.67	36.68	3.36
EUC_TP_334	22.13	5.62	11.37	0.67	31.10	3.35
EUC_TP_335	16.63	5.62	9.48	0.67	40.38	3.34

EUC_TP_336	19.85	5.62	12.37	0.67	29.91	3.34
EUC_TP_337	15.44	5.62	5.46	0.67	34.85	3.34
EUC_TP_338	26.17	5.62	20.94	0.67	40.33	3.23
EUC_TP_339	18.63	5.62	11.22	0.67	46.26	3.38
EUC_TP_340	22.84	5.62	11.01	0.67	42.02	3.34
EUC_TP_341	21.38	5.62	10.21	0.67	31.46	3.35
EUC_TP_342	21.50	5.62	10.27	0.67	34.40	3.23
EUC_TP_343	21.14	5.62	13.82	0.67	32.17	3.34
EUC_TP_344	30.77	5.62	18.84	0.67	29.11	3.33
EUC_TP_345	24.74	5.62	8.12	0.67	30.45	3.40
EUC_TP_346	22.23	5.62	10.92	0.67	45.13	3.34
EUC_TP_347	19.41	5.62	11.84	0.67	38.73	3.35
EUC_TP_348	24.22	5.61	11.11	0.67	43.92	3.36
EUC_TP_349	22.53	5.62	9.88	0.67	30.29	3.35
EUC_TP_350	15.85	5.61	3.48	0.67	46.01	3.35
EUC_TP_351	23.54	5.61	12.05	0.67	43.00	3.24
EUC_TP_352	5.06	5.61	0.64	0.67	44.90	3.36
EUC_TP_353	8.26	5.61	2.62	0.67	48.88	3.24
EUC_TP_354	4.97	5.62	0.44	0.67	43.18	3.35
EUC_TP_355	5.16	5.62	0.22	0.67	38.42	3.39
EUC_TP_356	0.71	5.62	0.65	0.67	39.85	3.39
EUC_TP_357	13.96	5.62	4.47	0.67	41.98	3.26
EUC_TP_358	5.86	5.62	1.20	0.67	37.90	3.37
EUC_TP_359	4.11	5.62	1.26	0.67	44.24	3.37
EUC_TP_360	0.80	5.61	0.01	0.67	36.99	3.37
EUC_TP_361	14.37	5.61	5.72	0.67	33.76	3.37
EUC_TP_362	19.90	5.62	12.24	0.67	33.29	3.34
EUC_TP_363	9.58	5.62	3.23	0.67	33.77	3.36
EUC_TP_364	15.26	5.61	6.26	0.67	43.74	3.35
EUC_TP_365	8.37	5.62	2.76	0.67	34.71	3.36
EUC_TP_366	12.16	5.62	2.36	0.67	48.93	3.34
EUC_TP_367	22.35	5.62	14.79	0.67	19.68	3.35
EUC_TP_368	24.86	5.62	11.13	0.67	33.96	3.37
EUC_TP_369	44.33	5.62	37.74	0.67	28.15	3.36
EUC_TP_370	29.39	5.62	18.00	0.67	29.74	3.34
EUC_TP_371	24.20	5.62	13.72	0.67	28.78	3.34
EUC_TP_372	39.23	5.62	33.55	0.67	28.28	3.35
EUC_TP_373	44.89	5.62	25.59	0.67	26.00	3.34
EUC_TP_374	34.04	5.62	22.22	0.67	27.68	3.35
EUC_TP_375	31.53	5.62	20.87	0.67	30.75	3.34
EUC_TP_376	25.43	5.62	18.13	0.67	28.81	3.35
EUC_TP_377	26.48	5.62	15.56	0.67	28.54	3.35
EUC_TP_378	23.63	5.61	10.62	0.67	30.50	3.34
EUC_TP_379	37.57	5.62	23.46	0.67	35.81	3.35
EUC_TP_380	35.01	5.62	15.97	0.67	34.32	3.35
EUC_TP_381	26.48	5.62	18.36	0.67	35.76	3.34
EUC_TP_382	31.52	5.61	21.67	0.67	29.66	3.34

EUC_TP_383	10.47	5.62	3.00	0.67	34.67	3.39
EUC_TP_384	31.90	5.62	16.79	0.67	32.18	3.35
EUC_TP_385	23.73	5.62	16.74	0.67	34.40	3.22
EUC_TP_386	38.65	5.62	30.65	0.67	28.78	3.35
EUC_TP_387	39.62	5.62	28.84	0.67	31.28	3.24
EUC_TP_388	31.56	5.62	17.77	0.67	34.86	3.35
EUC_TP_389	17.65	5.62	8.98	0.67	27.62	3.35
EUC_TP_390	45.24	5.62	28.50	0.67	29.72	3.35
EUC_TP_391	18.69	5.62	8.53	0.67	28.87	3.36
EUC_TP_392	12.50	5.61	5.25	0.67	34.12	3.36
EUC_TP_393	16.90	5.61	5.89	0.67	44.38	3.36
EUC_TP_394	20.84	5.62	14.60	0.67	37.95	3.35
EUC_TP_395	9.36	5.62	4.76	0.67	34.92	3.34
EUC_TP_396	16.78	5.61	3.52	0.67	34.33	3.36
EUC_TP_397	22.72	5.62	12.01	0.67	47.18	3.36
EUC_TP_398	42.09	5.62	28.03	0.67	41.14	3.23
EUC_TP_399	14.38	5.61	6.25	0.67	37.28	3.34
EUC_TP_400	8.74	5.61	0.35	0.67	28.72	3.34
EUC_TP_446	10.57	5.62	1.83	0.67	33.13	3.35
EUC_TP_447	31.04	5.62	6.87	0.67	32.87	3.53
EUC_TP_449	16.37	5.62	10.52	0.67	29.43	3.40
EUC_TP_454	8.57	5.62	1.50	0.67	26.28	3.26
EUC_TP_456	6.08	5.62	0.58	0.67	24.40	3.38
EUC_TP_495	68.99	5.62	59.27	0.67	NA	NA
EUC_TP_496	71.76	5.62	58.55	0.67	NA	NA
EUC_TP_497	67.12	5.62	53.62	0.67	NA	NA
EUC_TP_498	68.62	5.62	51.90	0.67	NA	NA
EUC_TP_499	79.92	5.61	73.48	0.67	NA	NA
EUC_TP_660	28.90	5.61	19.71	0.67	44.59	3.63
EUC_TP_661	7.52	5.62	0.01	0.67	43.29	3.36
EUC_TP_662	22.91	5.61	16.89	0.67	44.89	3.35

[a]square root transformed
[b]back transformed
